# Protocol to study the inheritance and propagation of non-genetically encoded states using barcode decay lineage tracing

**DOI:** 10.1016/j.xpro.2023.102809

**Published:** 2024-01-04

**Authors:** Yelyzaveta Shlyakhtina, Bianca Bloechl, Katherine L. Moran, Maximiliano M. Portal

**Affiliations:** 1 Cell Plasticity & Epigenetics Lab, Cancer Research UK Manchester Institute, The University of Manchester, Manchester M20 4BX, UK; 2 Cell Plasticity & Epigenetics Lab, Cancer Research UK – Cancer Research UK Scotland Institute, The University of Glasgow, Glasgow G61 1BD, UK

**Keywords:** Cell Biology, Genomics, Molecular Biology, Single Cell

## Abstract

Here, we present a protocol to perform barcode decay lineage tracing followed by single-cell transcriptome analysis (BdLT-Seq). We describe steps for BdLT-Seq experimental design, building barcoded episome reporters, performing episome transfection, and barcode retrieval. We then describe procedures for sequencing library construction while providing options for sample multiplexing and data analysis. This BdLT-Seq technique enables the assessment of clonal evolution in a directional manner while preserving isogeneity, thus allowing the comparison of non-genetic molecular features between isogenic cell lineages.

For complete details on the use and execution of this protocol, please refer to Shlyakhtina et al. (2023).^1^

## Highlights

• Transcriptome divergence can be studied in fully comparable (epi-)genomes over time
• Step-by-step generation of BdLT-Seq barcoded episome library by molecular cloning
• BdLT-Seq experimental design, episome transfection, and cell preparation
• 10× Chromium, BdLT-Seq reporter capture, NGS library preparation, and data analysis

## Abstract (editor highlights)

Publisher’s note: Undertaking any experimental protocol requires adherence to local institutional guidelines for laboratory safety and ethics.

## Graphical abstract

**Figure:**
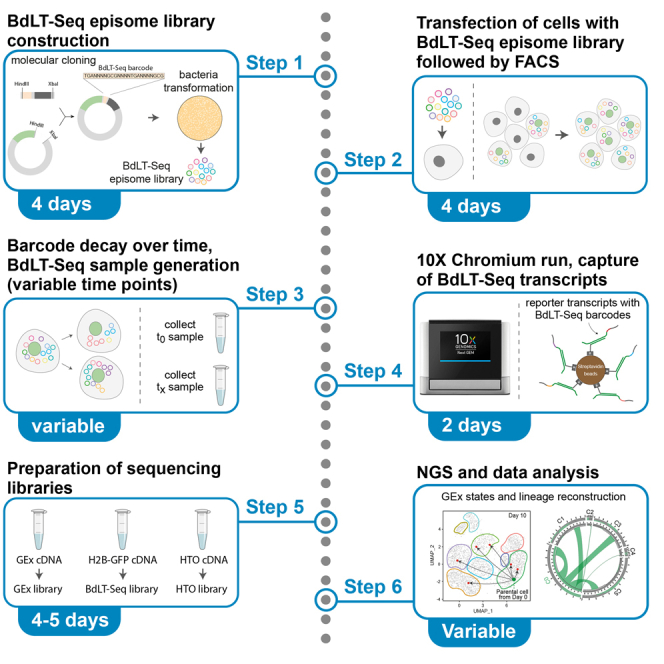

## Before you begin

Single-cell technologies are taking the world of biology research by storm as old concepts and frameworks are put to the challenge of multimodal single-cell analysis.^2^^,^^3^^,^^4^^,^^5^^,^^6^^,^^7^^,^^8^^,^^9^^,^^10^^,^^11^^,^^12^^,^^13^ It is in that view that multiple lines of research are converging onto the study of co-existing molecular states underlying a plethora of functional differences in cellular systems.^14^^,^^15^^,^^16^^,^^17^ Interestingly, the relationship between divergent molecular states becomes apparent as their dynamic nature could explain striking cellular features that remain elusive in multiple fields of biological research such as the emergence of drug resistance in cancer settings and phenotypic variation in stem cell reprogramming, among others.^18^^,^^19^^,^^20^^,^^21^ Strikingly, most of those features seem to be molecularly underpinned by the non-genetic compartment and, to a certain extent, controlled by it.^17^^,^^20^

**Figure 1:**
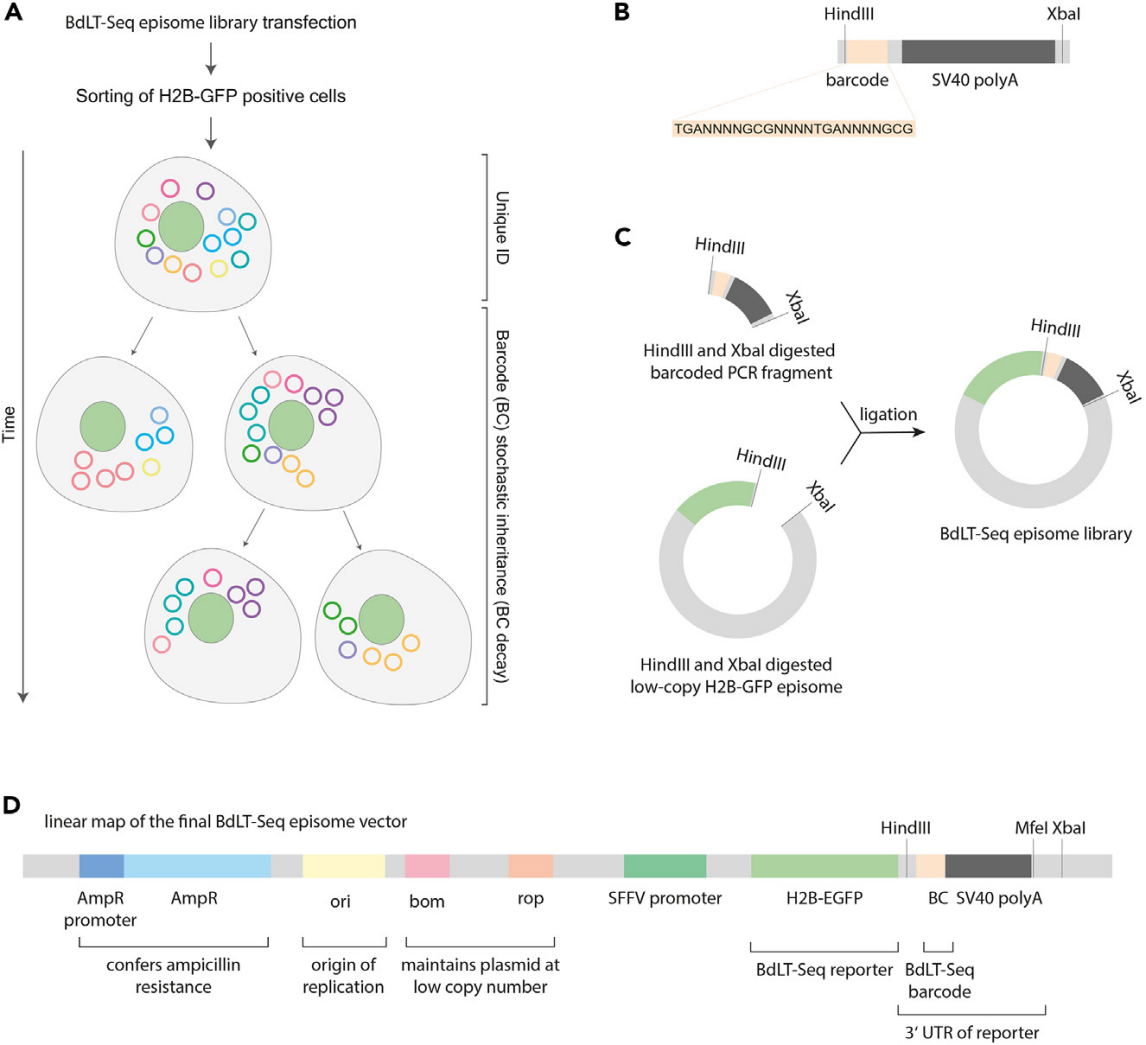
Development of Barcode decay Lineage Tracing (BdLT-Seq) (A) Scheme representing BdLT-Seq conceptual framework. H2B-GFP expression is used as a reporter molecule to enrich in episome containing cells by Fluorescence Activated Cell Sorting (FACS). Cell ID extraction and lineage tracing deconvolution is depicted as a function of time. (B) Scheme depicting barcoded fragment to be cloned into LT_vector episome backbone. (C) Scheme representing the BdLT-Seq episome library construction and ligation steps for its construction. (D) Schematic linear representation of the final BdLT-Seq episome.

It is worth stressing that state-of-the-art lineage tracing methodologies base their capability on the permanent modification of the host cell genome and thus reconstruct lineage trees mainly based on the sub-clonality of cells carrying either the same pool of genetically encoded tracers or genetically encoded tracers that evolve over time within the overall population.^22^^,^^23^^,^^24^^,^^25^^,^^26^^,^^27^^,^^28^^,^^29^^,^^30^^,^^31^ Moreover, due to the numerous divergent alterations introduced in the genome of the cells for their identification, the isogeneity within the population is lost and the phenotypic outputs cannot be unequivocally uncoupled from the genetic modifications introduced by the employed methodology. Therefore, in order to study the divergence of co-existing molecular states within a cellular population while minimizing the burden of potential method-introduced genotypic alterations within the studied cell/s, we built a robust lineage tracing platform that enables the study of co-existing cellular/molecular states and their divergence while maintaining cellular isogeneity at all times (Figure 1A), thus opening the door to the exploration of the role of the non-genetic compartment in cellular/molecular heterogeneity in genetically homogeneous cell populations.^1^

Briefly, BdLT-Seq brings a new facet to the lineage tracing field as the non-genetic nature of its molecular identifiers grants two key novel aspects; first, the dynamic decay of tracing information (barcodes, Figure 1A) upon cell division enables for the first time the assignment of experimentally-derived directionality to a cell lineage tree by linking every cell with its ancestor in a time-resolved manner; secondly, it maintains populational isogeneity allowing the comparison of non-genetic molecular features between isogenic cell-lineages. Indeed, BdLT-Seq is a modular and multimodal lineage tracing approach that can be readily combined with virtually any single cell platform (e.g., 10× Chromium), allowing the exploration of the molecular traits of individual cells within a population (e.g., transcriptome, epigenome) while providing unequivocal information about their identity and supporting the construction of robust directional lineage trees.

In this protocol, we make available to researchers a complete run-down of BdLT-Seq including information ranging from experimental design to data analysis. Every experimental step is depicted and annotated to help the researcher rapidly implement the method in their labs.

Before moving on to the actual protocol for BdLT-Seq, it is worth stressing that a critical part of it relies on the capture of barcoded H2B-GFP mRNA with the aid of strand-specific biotinylated probes. Prior to engaging the protocol, we strongly suggest the end-user to prepare the probes in advance as follows.

### Preparation of strand-specific biotinylated probes directed towards H2B-GFP mRNA

**Timing: 3 days**

This preparative step outlines the purification of four strand-specific biotinylated probes that will be used to capture H2B-GFP mRNA molecules in step 29 of the protocol. Probe sequences are provided in key resources table: LT_H2B-GFP_probes_Probe1-4, 0.05 μmol, cartridge-RP1 purified.

1. Probes must be purified via denaturing polyacrylamide gel electrophoresis (PAGE-Urea) following the protocol outlined below.
  a. Prepare 10% resolving gel:

**Table:** 

Reagent	For 2 gels
Urea (powder)	9.6 g
Bis-acrylamide 40% ratio 37.5/1 (Sigma, A7168)	4.5 mL
10× TBE	2 mL
ddH_2_O	To the final volume of 20 mL
APS 10%	160 μL
TEMED	24 μL

    **CRITICAL:** Bis-acrylamide is acutely toxic, may be carcinogenic and mutagenic and is suspected to damage fertility. When handling gels containing Bis-acrylamide, it is essential to exercise caution and to wear appropriate personal protective equipment. Always wear gloves when working with Bis-acrylamide solution or gels containing Bis-acrylamide.
    **CRITICAL:** APS is a health hazard, acute oral toxicity and irritation of skin and eyes. Oxidizing. Wear appropriate personal protective equipment.
    **CRITICAL:** TEMED is flammable and an acute health hazard when inhaled, swallowed or upon contact of skin or eyes. Wear appropriate personal protective equipment.
    **CRITICAL:** After combining urea, bis-acrylamide, TBE and ddH_2_O, mix the solution thoroughly by vortexing until the urea is completely dissolved. Then, add APS and TEMED while mixing.
  b. Load the gel and promptly gently cover it with 1 mL of ddH_2_O.
  c. Allow the gel to polymerize.
  d. Prepare stacking gel:

**Table:** 

Reagent	For 2 gels
Urea (powder)	4.8 g
Acrylamide/Bis-acrylamide, 40% solution, ratio 37.5:1	1 mL
10× TBE	1 mL
ddH_2_O	To the final volume of 10 mL
APS 10%	80 μL
TEMED	12 μL

    **CRITICAL:** please refer to resolving gel (step 1a)
    **CRITICAL:** After combining urea, bis-acrylamide, TBE and ddH_2_O, mix the solution thoroughly by vortexing until the urea is completely dissolved. Then, add APS and TEMED while mixing well.
  e. Before loading the stacking gel, carefully remove the ddH_2_O covering the resolving gel.
  f. Load the stacking gel and gently insert a suitable comb.
  g. Allow the gel to polymerize.
  h. Prepare the denaturing loading dye (2×):

**Table:** 

Reagent	Amount
10× TBE	1 mL
ddH_2_O	2.5 mL (final volume 10 mL)
Ficoll Type 400	1.2 g
Urea	4.2 g
Bromophenol Blue	8 mg (0.08% (w/v))
Xylene Cyanol FF	8 mg (0.08% (w/v))

    **CRITICAL:** Xylene Cyanol causes skin and eye irritations.
    ***Note:*** The loading dye can be prepared in a preparative manner and stored in aliquots at −20°C.
  i. Add denaturing loading dye to the probes (600 pmol per probe) in a 1:1 ratio and incubate at 95°C for 5 min to denature.
  j. Load probes onto the gel by loading 6 wells per individual probe at 100 pmol per well.
  k. Include a lane for a single-stranded DNA ladder (ssDNA ladder 20/100, IDT, cat. #51-05-15-02).
    **CRITICAL:** Ensure that empty wells are left between different samples and between samples and the DNA molecular weight ladder to prevent cross-contamination between wells.
  l. Perform gel electrophoresis at 120 V in 1× TBE buffer until the upper dye (Xylene Cyanol) reaches approximately the middle of the gel (20 cm gel).
  m. Switch off the current, remove the gel from the holder and retrieve it from between the glass plates.
  n. Stain the gel using SYBR Gold Nucleic Acid gel stain (Invitrogen, cat. #S11494) in 1× TBE buffer for 5 min.
2. Gel-purify the PAGE-separated probes.
  a. Place the gel onto a sheet of plastic wrap and illuminate it with a low power UV lamp transilluminator.
  b. Excise the bands displaying the expected molecular weight from the gel using a brand-new clean scalpel.
  c. Place the gel pieces into a 0.5 mL Eppendorf tube with a pierced bottom, then insert it into a 1.5 mL Eppendorf tube.
  d. Centrifuge for approximately 1 min at 16,900 × *g*. The gel slice will break into pieces as it passes through the Eppendorf tube.
  e. Add 600 μL of 1× NEBuffer 2 (NEB, cat. #B7002S) to the gel and incubate at room temperature (∼20°C) overnight (16 h) on a tube rotator to elute the DNA.
    ***Note:*** Ensure that the gel moves within the tube while shaking, otherwise complement with additional 100 μL of 1× NEBuffer 2.
  f. The next day, load the samples onto a Spin X Centrifuge Tube Filter 0.45 μm Cellulose Acetate 2 mL tube (Costar, cat. #8162) and centrifuge at 14,000 rpm for 2–5 min to filter the probes (liquid) from the gel.
  g. Collect the sample (∼400 μL) and transfer it into a fresh Eppendorf tube.
  h. Precipitate the probes by adding 40 μL of 3 M sodium acetate, 1–3 μL of Ethachinmate, and 1300 μL of absolute ethanol and mix well by inverting the tube.
  i. Incubate at −80°C overnight (16–24 h, or longer if required).
  j. Centrifuge the samples at 16,900 × *g* for 30 min at 4°C.
  k. Discard the supernatant and wash the DNA pellet with 85% ethanol.
  l. Centrifuge the samples once again at 16,900 × *g* for 10 min at 4°C.
  m. Discard the supernatant and air-dry the probes/DNA pellet.
  n. Resuspend and combine the pellets of each individual probe in a total of 15 μL of nuclease-free water per probe.
  o. Determine the probes/DNA concentration using a NanoDrop spectrophotometer (260 nm wavelength).
  p. Pool all four different probes in equimolar amounts to obtain a concentration of 4 pmol/μL or higher.
  q. Store the purified probes at −20°C.

## Key resources table

**Table:** 

REAGENT or RESOURCE	SOURCE	IDENTIFIER
**Antibodies**		

TotalSeq anti-human Hashtag 1 antibody, use at 10 μg/mL	BioLegend	Cat#A0251; clone LNH-94 2M2
TotalSeq anti-human Hashtag 2 antibody, use at 10 μg/mL	BioLegend	Cat#A0252; clone LNH-94 2M2
TotalSeq anti-human Hashtag 3 antibody, use at 10 μg/mL	BioLegend	Cat#A0253; clone LNH-94 2M2
TotalSeq anti-human Hashtag 4 antibody, use at 10 μg/mL	BioLegend	Cat#A0254; clone LNH-94 2M2
TotalSeq anti-human Hashtag 5 antibody, use at 10 μg/mL	BioLegend	Cat#A0255; clone LNH-94 2M2
TotalSeq anti-human Hashtag 6 antibody, use at 10 μg/mL	BioLegend	Cat#A0256; clone LNH-94 2M2
TotalSeq anti-human Hashtag 7 antibody, use at 10 μg/mL	BioLegend	Cat#A0257; clone LNH-94 2M2
TotalSeq anti-human Hashtag 8 antibody, use at 10 μg/mL	BioLegend	Cat#A0258; clone LNH-94 2M2
TotalSeq anti-human Hashtag 9 antibody, use at 10 μg/mL	BioLegend	Cat#A0259; clone LNH-94 2M2
TotalSeq anti-human Hashtag 10 antibody, use at 10 μg/mL	BioLegend	Cat#A0260; clone LNH-94 2M2

**Bacterial and virus strains**		

MegaX DH10B T1 bacteria strain	Invitrogen	Cat#C640003
SURE 2 supercompetent cells	Agilent Technologies	Cat#200152

**Chemicals, peptides, and recombinant proteins**		

XbaI	NEB	Cat#R0145
MfeI-HF	NEB	Cat#R3589
HindIII-HF	NEB	Cat#R3104
rCutSmart buffer	NEB	Cat# B6004S
Agarose UltraPure	Life Technologies	Cat#15510027
Ammonium persulfate	Sigma-Aldrich	Cat#215589
Expand high fidelity PCR system	Roche	Cat#11732641001
Trizma base	Sigma-Aldrich	Cat#RDD008
Acetic acid	Sigma-Aldrich	Cat#A6283
EDTA	Sigma-Aldrich	Cat#EDS
Gel loading dye (6×), blue	NEB	Cat#B7021
MinElute gel extraction kit	QIAGEN	Cat#28604
pGEM-T easy vector system	Promega	Cat#A1360
S.O.C. medium	Invitrogen	Cat#15544034
Ampicillin	Sigma-Aldrich	Cat#A5354
Plasmid Midi Kit	QIAGEN	Cat#12143
Ethidium bromide	Sigma-Aldrich	Cat#E1510
NEBNext Ultra II Directional RNA Library Prep Kit for Illumina	NEB	Cat#E7760
AMPure XP SPRI reagent	Beckman Coulter	Cat#A63880
Ethanol absolute	VWR	Cat#20821.365
Lipofectamine LTX reagent	Invitrogen	Cat#153385000
Opti-MEM	Invitrogen	Cat#31985062
Trypsin	Invitrogen	Cat#25300104
Fetal calf serum (FCS)	Gibco	Cat#A3/60801
Dimethyl sulfoxide (DMSO)	Sigma-Aldrich	Cat#D8418
Phosphate-buffered saline (PBS)	Sigma-Aldrich	Cat#P3818
Bovine serum albumin (BSA)	Sigma-Aldrich	Cat#A2153
Trypan blue	Invitrogen	Cat#T10282
xGen Hybridization and Wash Kit	IDT	Cat#1080577
Dynabeads MyOne Silane beads	Invitrogen	Cat#37002D
RLT lysis buffer	QIAGEN	Cat#79216
Boric acid	Sigma-Aldrich	Cat#B6768
1 kb DNA ladder	NEB	Cat#N3232
100 bp DNA ladder	NEB	Cat#N3231
ssDNA ladder 20/100	IDT	Cat#51-05-15-02
Nuclease-free water: UltraPure distilled water (DNase/RNase-free)	Invitrogen	Cat#10977-035
NEBuffer 2	NEB	Cat#B7002S
SYBR Gold nucleic acid gel stain	Invitrogen	Cat#S11494
Urea, powder	Sigma-Aldrich	Cat#U5378
N,N,N′,N′-tetramethylethylenediamine (TEMED)	Sigma-Aldrich	Cat#T9281
Ficoll 400	Sigma-Aldrich	Cat#F8016
Bromophenol blue	Sigma-Aldrich	Cat#114391
Xylene cyanol FF	Sigma-Aldrich	Cat#335940
Ammonium persulfate (APS)	Sigma-Aldrich	Cat#A3678
Acrylamide/Bis-acrylamide, 40% solution, ratio 37.5:1	Sigma-Aldrich	Cat#A7168
Acetic acid	Sigma-Aldrich	Cat#A6283
Tris base	Sigma-Aldrich	Cat#RDD008
Ethylenediaminetetraacetic acid disodium salt dihydrate (EDTA)	Sigma-Aldrich	Cat#E5134
Sodium hydroxide	Honeywell	Cat#S8045
Ethachinmate	NIPPON GENE	Cat#321-01791
Sodium acetate solution, 3 M	Sigma-Aldrich	Cat#71196
MiSeq Reagent Micro Kit v2	Illumina	Cat#MS-103-1002
Dual Index Kit TT Set A	10× Genomics	Cat#1000215
Qubit RNA Quantification Assay Kits, RNA high sensitivity	Invitrogen	Cat#Q32852
Qubit dsDNA Quantification Assay Kits, dsDNA broad range	Invitrogen	Cat#Q32850
Qubit dsDNA Quantification Assay Kits, dsDNA high sensitivity	Invitrogen	Cat#Q32851
Qubit assay tubes	Invitrogen	Cat#Q32856

**Critical commercial assays**		

Chromium Next GEM Single Cell 3ʹ Kit v3.1 (Single Cell 3′ Library & Gel Bead Kit v3.1 (Dual Index))	10× Genomics	Cat#1000269
Chromium Chip G Single Cell Kit	10× Genomics	Cat#1000127

**Deposited data**		

BdLT-Seq as a barcode decay-based method to unravel lineage-linked transcriptome plasticity	GEO (Gene Expression Omnibus)	GEO: GSE223496

**Experimental models: Cell lines**		

HA1ER	William C. Hahn Lab (Dana-Farber Cancer Institute)	N/A
HA1E	William C. Hahn Lab (Dana-Farber Cancer Institute)	N/A

**Oligonucleotides**		

LT_H2B-GFP_barcode_fragment (forward primer) ACGAGCAAGCTTAG TAATGANNNNGCGNNNNTGANNNN GCGGATCCAGACATGATAAGATACA TTGATGAGTTTGG	Merck	N/A
LT_H2B-GFP_barcode_fragment (reverse primer) GATCGATCCTCTA GAGTCGACCTCAT	Merck	N/A
LT_H2B-GFP_probes_Probe1 [BTN]GTT CAGCGTGTCCGGCGAGGGCGAGGG CGATGCCACCTACGGCAAGCTGACC CTGAAGTTCATCTGCACCACCGGCAAGC	Merck	0.05 μmol, cartridge-RP1 purification
LT_H2B-GFP_probes_Probe2 [BTN]CAGCACGACTTCTTCAA GTCCGCCATGCCCGAAGGCT ACGTCCAGGAGCGCACCATC TTCTTCAAGGACGACGGCAACTA	Merck	0.05 μmol, cartridge-RP1 purification
LT_H2B-GFP_probes_Probe3 [BTN]ACGGCAACATCCTGGGGC ACAAGCTGGAGTACAACTACAA CAGCCACAACGTCTATATCATG GCCGACAAGCAGAAGAAC	Merck	0.05 μmol, cartridge-RP1 purification
LT_H2B-GFP_probes_Probe4 [BTN]CCCCATCGGCGACGGCC CCGTGCTGCTGCCCGACAACC ACTACCTGAGCACCCAGTCCG CCCTGAGCAAAGACCCCAACG	Merck	0.05 μmol, cartridge-RP1 purification
LT_H2B-GFP_library_Forward primer_BC1 CAAGCAGAAGACG GCATACGAGATCGTGATGTGAC TGGAGTTCAGACGTGTGCTCTT CCGATCTCTCGGCATGGACGA GCTGTACAAGT	Merck	N/A
LT_H2B-GFP_library_Forward primer_BC2 CAAGCAGAAGACGG CATACGAGATGCCTAAGTGACTG GAGTTCAGACGTGTGCTCTTCCG ATCTCTCGGCATGGACGAGCTGT ACAAGT	Merck	N/A
LT_H2B-GFP_library_Forward primer_BC3 CAAGCAGAAGACGGCATA CGAGATTGGTCAGTGACTGGAGTTCA GACGTGTGCTCTTCCGATCTCTCGGCA TGGACGAGCTGTACAAGT	Merck	N/A
LT_H2B-GFP_library_Reverse primer AATGATACGGCGACCACCGA GATCTACACTCTTTCCCTACACGACG CTCTTCCGATCT	Merck	N/A
Primers for construction of NEBNext libraries, HTO libraries and HTO additive, see Table S1	Merck	N/A

**Recombinant DNA**		

LTv-H2B-GFP (in this paper referred to as LT_vector)	Shlyakhtina et al.^1^	N/A
LTv-BC-H2B-GFP episome library (in this paper referred to as BdLT-Seq episome library)	Shlyakhtina et al.^1^	N/A

**Software and algorithms**		

FlowJo software v10.8.0	BD	N/A
R v4.2.0	N/A	https://www.r-project.org/
Cutadapt v3.7	Martin et al.^32^	https://github.com/marcelm/cutadapt
CITE-seq-count v1.4.5	Stoeckius et al.^33^	https://github.com/Hoohm/CITE-seq-Count
Cell Ranger v7.1.0	10× Genomics	https://github.com/10XGenomics/cellranger
Barcode similarity pipeline v1.0.0	Shlyakhtina et al.^1^	https://static-content.springer.com/esm/art%3A10.1038%2Fs41467-023-36744-1/MediaObjects/41467_2023_36744_MOESM4_ESM.txt
Seurat v4 or later	Butler et al.^34^ and Stuart et al.^35^	https://satijalab.org/seurat/
CIRCOS	Krzywinski et al.^36^	http://circos.ca/

**Other**		

Sub-cell GT horizontal electrophoresis system, 15 × 20 cm tray	Bio-Rad	Cat#1704403
Benchtop refrigerated centrifuge for 1.5–2 mL	Eppendorf	Cat#5418R
Refrigerated centrifuge 5810 R	Eppendorf	Cat#5811000061
Bio-Rad Molecular Imager Gel Doc XR system	Bio-Rad	Cat#170-8170
Tube rotator Loopster basic	IKA	Cat# 0004015000
ProFlex PCR system	Applied Biosystems	Cat#4484073
Vortex mixer	Fisherbrand	Cat#11746744
0.1 cm electroporation cuvette	Bio-Rad	Cat#1652089
MicroPulser electroporator	Bio-Rad	Cat#1652100
L-shaped cell spreader	Fisherbrand	Cat#14-665-230
Fragment analyzer 5200	Agilent	Cat#M5310AA
CO_2_ cell culture incubator	PHCbi	Cat#MCO-170AICUVH
BD LSRFortessa X-20 cell analyzer	BD Biosciences	N/A
BD FACSAria III cell sorter	BD Biosciences	N/A
BD Influx cell sorter	BD Biosciences	N/A
10× Chromium controller	10× Genomics	Cat#1000204
ThermoMixer C	Eppendorf	Cat#EP5382000015
Mini-PROTEAN Tetra vertical electrophoresis for handcast gels	Bio-Rad	Cat#1658000EDU
Incubator shaker	INFORS HT Multitron	Cat#2292113-18
Tissue culture plate, 6 well	Falcon	Cat#353047
Tissue culture plate, 24 well	Falcon	Cat#353072
Tissue culture plate, 96 well	Falcon	Cat#353046
Tissue culture dish, 100 mm × 20 mm	Falcon	Cat#353003
Neubauer counting chamber	Marienfeld	Cat#0640030
Spin-X centrifuge tube filter 0.45 μm cellulose acetate 2 mL tube	Costar	Cat#8162
DynaMag2	Invitrogen	Cat#12321D
NovaSeq 6000 version 1.5	Illumina	Cat#20013850
MiSeq system	Illumina	Cat#SY-410-1003
NanoDrop One microvolume UV-vis spectrophotometer	Thermo Fisher Scientific	Cat#ND-ONE-W
Qubit 3.0 fluorometer	Thermo Fisher Scientific	Cat#Q33216
5200 Fragment analyzer	Agilent	Cat#M5310AA

## Materials and equipment

**Table:** EDTA 0.5 M pH 8.0

Reagent	Final concentration	Amount
EDTA disodium salt dihydrate	0.5 M	93.06 g
Sodium hydroxide (NaOH)	N/A	To pH 8.0
ddH_2_O	N/A	To 500 L
**Total**	**N/A**	**0.5 L**

Store at room temperature (∼20°C), shelf life one year.

**Table:** 50× TAE buffer

Reagent	Final concentration	Amount
Tris base	2 M	242 g
Acetic Acid (100%)	1 M	57.1 mL
EDTA pH 8.0 (0.5 M)	50 mM	100 mL
ddH_2_O	N/A	To 1 L
**Total**	**N/A**	**1 L**

Store at room temperature (∼20°C), shelf life one year.

**Table:** 10× TBE buffer

Reagent	Final concentration	Amount
Tris base	0.9 M	108 g
Boric Acid	0.9 M	55 g
EDTA pH 8.0 (0.5 M)	20 mM	40 mL
ddH_2_O	N/A	To 1 L
**Total**	**N/A**	**1 L**

Store at room temperature (∼20°C), shelf life one year.

• **0.04% (w/v) BSA PBS solution:** add 0.02 g BSA in 50 mL 1x PBS buffer. Filter and keep at 4°C. Use on the same day.
• **5% (v/v) FCS PBS solution:** add 2.5 mL FCS in 50 mL 1x PBS buffer. Filter and keep at 4°C. Use on the same day.
• **Denaturation Buffer:** 100 mM NaOH, 0.1 mM EDTA. Filter, keep at room temperature (∼20°C) and use within a week.

## Step-by-step method details

### Generate a barcoded lineage tracing vector library

**Timing: 4 days**

This step outlines the cloning strategy necessary to create a complex, ready-to-use library of barcoded episomes (LT_vector; Figure 1D) that can then be utilized to conduct Barcode decay Lineage Tracing experiments. The LT_vector (low-copy number in bacteria + H2B-GFP mammalian expression episome) is used as a template to generate a barcoded fragment which will be subsequently cloned back into the template LT_vector (Figures 1B and 1C) resulting in a high-complexity barcoded lineage tracing episome library (Figure 1D).

1. To this end, amplify a fragment of the 3′UTR of the H2B-GFP in the LT_vector by Polymerase Chain Reaction (PCR) using the Expand High Fidelity PCR System (Roche, cat. #11732641001) and a set of primers (see table below) where the forward primer contains a 12 nucleotide long random sequence split into three 4 nt long segments (NNNN) by intervening/anchoring nucleotides (GCG and TGA; final primer: NNNNGCGNNNNTGANNNN).
  ***Note:*** To increase the multiplicity of barcodes present within the final sample we recommend performing several PCR reactions (3–4) to ensure sufficient material is generated for the subsequent steps, which can be combined during gel purification of the amplified barcoded fragment.
  a. Prepare the PCR reaction mix as follows:

**Table:** 

Reagent	Amount per reaction
LT_vector episome (20 ng/ μL)	5 μL
Forward primer containing barcodes: LT_H2B-GFP_barcode_fragment (Fwd) (20 μM)	1 μL
Reverse primer: LT_H2B-GFP_barcode_fragment (Rev) (20 μM)	1 μL
dNTPs (10 mM each)	1 μL
Expand High Fidelity Enzyme mix	0.75 μL
Expand High Fidelity Buffer (10×) with 15 mM MgCl_2_	5 μL
Nuclease-free water	36.25 μL
  b. Thoroughly mix by pipetting up and down at least 10 times, then proceed with PCR amplification as follows.

**Table:** PCR cycling conditions:

Steps	Temperature	Time	Cycles
Initial Denaturation	94°C	2 min	1
Denaturation	94°C	15 s	36 cycles
Annealing	62°C	30 s	
Extension	72°C	45 s (+5 s in every cycle starting from cycle 11)	
Final extension	72°C	7 min	1
Hold	4°C

    **Pause point:** Proceed to the next step or freeze the PCR product at −20°C.
    ***Note:*** When using the Expand High Fidelity PCR System, the gradual increase of the extension time from cycle 11 onwards provides increased yields of the amplified product and increased proof-reading activity during the final phases of the amplification.
2. Resolve the PCR amplified fragments by apparent molecular weight using agarose gel electrophoresis.
  a. To do this, prepare 300 mL of a 1.4% (w/v) agarose solution in 1× TAE buffer.
    i. After melting the agarose in a microwave, allow it to cool to ∼60°C.
    ii. Add 3 μL of Ethidium Bromide (10 mg/mL) and mix thoroughly by swirling.
    iii. pour the mixture into a 20 cm gel tray, insert a suitable size comb (estimate 45 μL per well) and let the gel set.
      **CRITICAL:** Ethidium Bromide is a mutagen and carcinogen. Therefore, cautious handling is necessary when dealing with gels containing this compound. Always wear gloves when working with Ethidium Bromide solution or gels that contain it. Opt for Ethidium Bromide solution instead of the powder form or use an alternative DNA dye such as SYBR Safe or Gel Red. Please use a plastic wrap when positioning the gel in the UV apparatus to avoid potential contamination.
  b. Load and run the agarose gel.
    i. Combine 50 μL of each PCR reaction with 10 μL of 6× DNA loading dye.
    ii. After the gel has set, load 40–50 μL of each PCR reaction in each well.
    iii. In a different well, load a double-stranded DNA molecular weight ladder (100 bp).
      **CRITICAL:** Ensure that wells are left empty between different samples and between samples and the DNA ladder. This precaution is essential to prevent cross-contamination between samples of different origin.
    iv. Conduct gel electrophoresis at 150 V in 1× TAE buffer until the lower dye (bromophenol blue) migrates to approximately 5 cm from the bottom of the gel.
    v. After switching off the current, take the gel out of the tray, lay it on a sheet of plastic wrap, and expose it to UV illumination from a transilluminator at low lamp power.
      ***Note:*** Ensure that gel electrophoresis is performed for a suitable duration to properly separate the required DNA molecules on a case-by-case basis depending on the molecular weight of the DNA fragments that the user wishes to separate. Insufficient gel migration might compromise the integrity and purity of the required material after DNA purification.
      ***Note:*** Ethidium Bromide is positively charged and migrates towards the cathode, while the DNA sample migrates towards the anode. As gel electrophoresis proceeds, Ethidium Bromide gradually moves out of the gel, resulting in reduced fluorescent intensity towards the gel's end.
3. Gel-purify the PCR-amplified barcoded fragment.
  a. Excise a gel slice of approximately 3–4 mm in size, corresponding to the expected molecular weight of the barcoded fragment (459 bp).
  b. Use the MinElute Gel Extraction Kit (QIAGEN, cat. #28604) to purify the PCR-amplified barcoded fragment from the gel, following the manufacturer’s instructions.
    i. Use 20 μL of nuclease-free water to elute the barcoded fragment DNA from the columns.
      ***Note:*** Multiple gel slices from multiple PCR reactions of the barcoded fragment can be pooled into one purification reaction.
  c. Evaluate recovery of the gel-purified barcoded fragment by Qubit quantification.
    ***Note:*** Alternatively, barcoded fragment DNA can be quantified using a NanoDrop Spectrophotometer (260 nm wavelength).
    **Pause point:** Store the purified barcoded fragment DNA at −20°C or proceed to the next step.
4. Carry out parallel restriction digestion reactions on the LT_vector episome and the barcoded fragment using HindIII-HF (NEB, cat. #R3104) and XbaI (NEB, cat. #R0145) restriction enzymes following the manufacturer’s instructions.
  a. Briefly, add 10–20 U of each restriction enzyme per 1 μg DNA in 1× CutSmart buffer.
  b. Incubate at 37°C for 15–60 min.

***Note:*** To generate enough material to perform multiple ligation reactions and bacterial transformations in subsequent steps, we recommend performing several restriction digest reactions for both the LT_vector and the PCR-amplified barcoded fragment. These can be combined during gel purification.

5. Perform agarose gel electrophoresis to separate by molecular weight the DNA fragments obtained from the restriction digestions.
  ***Note:*** and purify both the barcoded fragment and the backbone LT_vector where the barcoded fragment is going to be introduced. To ensure proper separation of both required DNA fragments, two gels of different agarose concentration must be prepared, one for the barcoded amplicon (1.4% w/v agarose) and another for the backbone episome (0.8% w/v agarose).
  a. Prepare 300 mL of a 0.8% (w/v) and 300 mL of a 1.4% (w/v) agarose solution in 1× TAE buffer.
    i. After melting the agarose in a microwave, allow it to cool to ∼60°C.
    ii. Add 3 μL of Ethidium Bromide (10 mg/mL) to each preparation and mix thoroughly by swirling.
    iii. Pour each mixture into a 20 cm gel tray and then insert a suitable size comb (estimate 45 μL per well) and let the gel set.
      **CRITICAL:** See step 2a.
  b. Load and run the agarose gels.
    i. Combine 50 μL of the restriction digestion reaction with 10 μL of 6× DNA loading dye.
    ii. After the gels have set, load 40–50 μL of the digested barcoded fragment per well into the 1.4% agarose gel and 40–50 μL of the digested LT_vector per well into the 0.8% agarose gel.
    iii. Include a well containing a double-stranded DNA ladder, specifically using a 100 bp ladder for the 1.4% agarose gel (barcoded fragment) and a 1 kb ladder for the 0.8% agarose gel (LT_vector).
      **CRITICAL:** Ensure that wells are left empty between different samples and between samples and the DNA ladder. This precaution is essential to prevent cross-contamination between samples of different origin.
    iv. Conduct gel electrophoresis at 150 V in 1× TAE buffer until the lower dye (bromophenol blue) migrates to approximately 5 cm from the bottom of the gel.
    v. After switching the current off, take the gel out of the tray, lay it on a sheet of plastic wrap, and expose it to UV illumination from a transilluminator at low lamp power.
      ***Note:*** see step 2b.
6. Gel-purify the digested barcoded fragment and the digested LT_vector template.
  a. Excise a gel slice of approximately 3–4 mm in size, corresponding to the expected molecular weight (LT_vector episome – 8409 bp, barcoded fragment – 438 bp).
  b. Use the MinElute Gel Extraction Kit (QIAGEN, cat. #28604) to purify both the digested barcoded amplicon and the LT_vector template from their respective gels, following the manufacturer’s instructions.
    i. Elute both samples in 20 μL of nuclease-free water.
      ***Note:*** Multiple gel slices from multiple reactions of the same restriction digest can be pooled into one purification reaction.
  c. Quantify obtained DNA using Qubit fluorometric quantification.
    ***Note:*** Alternatively, DNA quantification can be performed using a NanoDrop Spectrophotometer (260 nm wavelength).
7. Ligate the purified barcoded fragment, digested with HindIII and XbaI, into the LT_vector episome which, as it has also been digested with HindIII and XbaI, bears compatible ends, using T4 DNA ligase from the pGEM-T easy vector system (Promega, cat. #A1360).
  a. Ligation reaction:

**Table:** 

Reagent	Amount/tube
Rapid Ligation Buffer (2×)	5 μL
LT_vector episome	300 ng
Barcoded fragment DNA	64 ng
Nuclease-free water	To 10 μL total volume
T4 DNA Ligase	1 μL
  b. Thoroughly mix by pipetting up and down 10 times.
  c. Incubate the ligation reaction at 4°C overnight (∼16 h).
    ***Note:*** When performing multiple ligation reactions, a master mix can be prepared but the reaction mix should be aliquoted with 10 μL per tube for overnight incubation at 4°C.
    **CRITICAL:** Do not heat-inactivate the T4 DNA ligase prior to bacterial transformation as this substantially decreases transformation efficiency.
8. Perform bacterial transformation using the electrocompetent MegaX DH10B T1 strain (Invitrogen, cat. #C640003), following the manufacturer’s instructions.
  a. Briefly, thaw the bacteria on ice for approximately 5 min.
  b. Add 8 μL of the ligation reaction from the previous step to 100 μL of bacteria.
    ***Note:*** When performing multiple transformation reactions in parallel, the ligation reactions can be pooled and 8 μL used per transformation reaction of 100 μL bacteria. For example, 5 ligation reactions (50 μL in total) can be used for 6 transformation reactions.
  c. Gently mix by flicking and transfer the mixture into a pre-chilled 0.1 cm electroporation cuvette (Bio-Rad, cat. #1652089).
  d. Electroporate using the Ec1 program on the MicroPulser Electroporator (Bio-Rad, cat. #1652100).
  e. Immediately after, add 1 mL of pre-warmed S.O.C. medium to the cuvette and transfer the contents to a microcentrifuge tube.
  f. For the recovery process, incubate the bacteria at 37°C in an incubator shaker (Infors, HT Multitron, cat. #2292113-18) at 225 rpm for 1 h.
  g. After incubation, collect the bacteria through centrifugation at 400–600 × *g*, then resuspend the bacterial pellet in 100 μL LB medium.
  h. Plate serial dilutions (1/10 to 1/10,000) onto 10 cm LB agar plates containing 50 mg/mL ampicillin and plate approximately 40–45 μL per plate (resulting in two undiluted plates per electroporation).
  i. Incubate bacterial plates overnight (∼16 h) at 37°C.
    **CRITICAL:** Avoid processing too many bacteria transformations simultaneously, we recommend not processing more than 4 transformations in parallel. The time taken both before and after bacteria electroporation is critical for optimal recovery post electroporation. Spending a prolonged amount of time before and after electroporation will significantly reduce the transformation efficiency. When performing multiple transformation reactions in parallel, add pre-warmed S.O.C medium immediately after the electroporation before continuing with the next electroporation to improve recovery.
    ***Note:*** Alternative equipment can be used for bacterial electroporation.
    ***Note:*** The number of colonies obtained where serial dilutions were plated can be used to estimate the maximum number of barcodes in the undiluted plates.
    **CRITICAL:** The plates generated by plating undiluted bacteria are meant for collection and plasmid extraction. Plating between 30 K-300 K bacteria per plate inhibits colony growth, thereby reducing the number of episome copies per colony and, therefore, reducing the number of identical barcodes. Keeping the multiplicity of barcodes very low is crucial for lineage tracing experiments described in the next steps.
    **CRITICAL:** Do not allow bacterial plates to overgrow. Collect colonies from undiluted plates where small colonies (<1 mm) are observed. If required, incubation temperature can be reduced to 30°C to slow down colony growth. This step is critical to keep barcode multiplicity low.
    ***Note:*** Multiple rounds of transformation might be needed to obtain sufficient barcode heterogeneity (number of unique barcodes). We typically aim to obtain between 0.5 × 10^6^ to 1 × 10^6^ bacteria colonies in total (i.e. unique barcodes estimate).
    **CRITICAL:** We have observed significant variability in the transformation potential of electrocompetent bacteria across different batches (MegaX DH10B T1 strain, Invitrogen, cat. #C640003). Therefore, if bacteria transformation is underperforming, we recommend to immediately replace the batch to a brand new one to improve transformation efficiency.
9. Collect bacterial colonies from the LB plates generated by plating undiluted bacteria.
  a. To do this, add 5 mL of LB medium per plate.
  b. Scrape the bacterial colonies from the agar surface and transfer to a centrifuge tube.
  c. Wash the plate twice with 5 mL of LB medium to collect all remaining bacteria.
  d. Collect bacteria through centrifugation at 6,000 × *g* for 20 min at 4°C and dispose of the supernatant.

**Pause point:** Freeze the bacteria pellet at −80°C or proceed to the next step.

10. Extract plasmid DNA (BdLT-Seq episome library) using the Plasmid Midi Kit (QIAGEN, cat. #12143) following the manufacturer’s instructions.
11. Quantify obtained DNA using Qubit fluorometric quantification.

***Note:*** Alternatively, DNA quantification can be performed using a NanoDrop Spectrophotometer (260 nm wavelength).

**Pause point:** Store the purified BdLT-Seq episome library at −20°C.

### Assess barcode multiplicity of the BdLT-Seq library using NGS

**Timing: 3 days plus sequencing processing time**

In this step of the protocol, the number of unique barcodes and barcode multiplicity within the generated BdLT-Seq episome library is assessed using Next Generation Sequencing.

12. Digest 5 μg of BdLT-Seq episome library using HindIII-HF (NEB, cat. #R3104) and MfeI-HF (NEB, cat. #R3589) restriction enzymes (see Figure 1D), following the manufacturer’s instructions.
  a. Briefly, add 10–20 U of each restriction enzyme per 1 μg DNA in 1× CutSmart buffer.
  b. Incubate at 37°C for 15–60 min.
13. To identify and purify the barcoded fragment, perform agarose gel electrophoresis to separate the resulting DNA molecules obtained after restriction digestion.
  a. To do this, prepare 300 mL of a 2% (w/v) agarose solution in 1× TAE buffer.
    i. After melting the agarose in a microwave, allow it to cool to ∼60°C.
    ii. Add 3 μL of Ethidium Bromide (10 mg/mL) and mix thoroughly by swirling.
    iii. Pour the mixture into a 20 cm gel tray, and then insert a suitable size comb (estimate 45 μL per well) and let the gel set.
      **CRITICAL:** Ethidium Bromide is a mutagen and carcinogen. Therefore, cautious handling is necessary when dealing with gels containing this compound. Always wear gloves when working with Ethidium Bromide solution or gels that contain it. Opt for Ethidium Bromide solution instead of the powder form or use an alternative DNA dye such as SYBR Safe or Gel Red. Please use a plastic wrap when positioning the gel in the UV apparatus to avoid potential contamination.
  b. Load and run the agarose gel.
    i. Combine 50 μL of the restriction digestion reaction with 10 μL of 6× DNA loading dye.
    ii. After the gel has set, load 40–50 μL of this mixture into each well.
    iii. Include a well containing a double-stranded DNA molecular weight ladder (100 bp).
      **CRITICAL:** Ensure that empty wells are left between different samples and between samples and the DNA molecular weight ladder.
    iv. Conduct gel electrophoresis at 150 V in 1× TAE buffer until the lower dye (bromophenol blue) migrates to approximately 5 cm from the bottom of the gel.
    v. After switching off the current, take the gel out of the tray, lay it on a sheet of plastic wrap, and expose it to UV illumination from a transilluminator at low lamp power.
      ***Note:*** Ensure that gel electrophoresis is performed for a suitable duration to properly separate the required DNA molecules on a case-by-case basis depending on the molecular weight of the DNA fragments that the user wishes to separate. Insufficient gel migration might compromise the integrity and purity of the required material after DNA purification.
      ***Note:*** Ethidium Bromide is positively charged and migrates towards the cathode, while the DNA sample migrates towards the anode. As gel electrophoresis proceeds, Ethidium Bromide gradually moves out of the gel, resulting in reduced fluorescent intensity towards the gel's end.
14. Gel-purify the digested barcoded fragment.
  a. Excise a gel slice of approximately 3–4 mm in size, corresponding to the expected molecular weight (barcoded fragment – 178 bp).
  b. Use the MinElute Gel Extraction Kit (QIAGEN, cat. #28604) to purify the restriction digested barcoded fragment from the gel, following manufacturer’s instructions.
    i. Elute DNA in 20 μL of nuclease-free water.
  c. Quantify obtained DNA using Qubit fluorometric quantification.

***Note:*** Alternatively, you can perform DNA quantification using a NanoDrop spectrophotometer (260 nm wavelength).

**Pause point:** Proceed to the next step or freeze purified DNA at −20°C.

15. Use 18–20 ng of purified DNA to prepare an Illumina-compatible sequencing library using NEBNext Ultra II Directional RNA Library Prep Kit for Illumina (NEB, cat. #E7760).
  a. Perform End repair 5′ phosphorylation and A-tailing reaction.
    i. End preparation:

**Table:** 

Reagent	Amount/tube
Purified barcoded fragment	18–20 ng
TE buffer	to 60 μL final volume
NEBNext Ultra II End Prep Reaction Buffer	7 μL
NEBNext Ultra II End Prep Enzyme Mix	3 μL
    ii. Thoroughly mix by pipetting up and down 10 times, and then incubate in a thermocycler as follows:

**Table:** 

Temperature	Time
20°C	30 min
65°C	30 s
4°C	Hold

Lid temperature ≥ 75°C
  b. Perform adaptor ligation.
    i. Prepare a 5-fold dilution of the supplied adaptor (NEB, cat. #E7760).
    ii. Proceed with the ligation reaction:

**Table:** 

Reagent	Amount/tube
Sample	60 μL
Diluted adaptor	2.5 μL
NEBNext Ligation Enhancer	1 μL
NEBNext Ultra II Ligation Master Mix	30 μL
    iii. Thoroughly mix by pipetting up and down 10 times, then incubate for 15 min at 20°C.
    iv. Add 3 μL of USER enzyme, mix thoroughly, and incubate for 15 min at 37°C.
  c. Purify ligated material using SPRI beads (Beckman Coulter, AMPure XP, cat. #A63880).
    i. Resuspend SPRI beads thoroughly by vortexing before use.
    ii. Add 87 μL (0.9×) of resuspended SPRI beads to the sample and mix thoroughly by pipetting up and down at least 10 times.
    iii. Incubate for 10 min at room temperature (∼20°C).
    iv. Place the tube on a magnet.
    v. Once the solution is clear, discard the supernatant.
    vi. Wash twice by adding 200 μL of 80% ethanol to the tube while it’s on the magnetic rack (DynaMag2; Invitrogen, cat. #12321D) and incubate at room temperature (∼20°C) for 30 s.
    vii. After the second wash, briefly spin the tube and return it to the magnetic rack.
    viii. Remove any remaining ethanol, and allow beads to air dry.
      **CRITICAL:** Avoid excessive drying of SPRI beads, as this reduces recovery efficiency. Elute samples when beads appear dark brown and glossy.
    ix. Elute DNA from the SPRI beads by adding 17 μL of TE buffer (NEB, cat. #E7760) and mix thoroughly by pipetting up and down at least 10 times.
    x. Incubate beads for 2 min at room temperature (∼20°C).
    xi. Place the tube back onto the magnetic rack and once separation is complete, transfer 15 μL of the supernatant into a fresh tube.
      **Pause point:** If required, samples can be stored at −20°C.
  d. Perform PCR enrichment of the adaptor-ligated DNA.
    i. PCR reaction:

**Table:** 

Reagent	Amount/tube
Adaptor ligated DNA	15 μL
NEBNExt Ultra II Q5 Master Mix	25 μL
NEBNext_index_primer_for_Illumina (10 μM)	5 μL
NEBNext_universal_PCR_primer_for_Illumina (10 μM)	5 μL

      ***Note:*** If sample multiplexing is required, please refer to the corresponding NEBNext Oligo kit manual (e.g. for NEB cat. #E7335) to determine valid barcode combinations.
    ii. Thoroughly mix by pipetting up and down at least 10 times, then proceed with PCR amplification in a benchtop thermocycler as follows:

**Table:** 

Steps	Temperature	Time	Cycles
Initial Denaturation	98°C	30 s	1
Denaturation	98°C	10 s	5 cycles
Annealing and Extension	65°C	75 s	
Final extension	65°C	5 min	1
Hold	4°C
  e. Purify the PCR product (barcoded BdLT-Seq library fragment) using SPRI beads
    i. Resuspend SPRI beads thoroughly by vortexing.
    ii. Add 45 μL (0.9×) of resuspended SPRI beads and mix thoroughly by pipetting up and down at least 10 times.
    iii. Incubate for 5 min at room temperature (∼20°C) and then place the tube on a magnet.
    iv. Once the solution is clear, discard the supernatant.
    v. Wash twice by adding 200 μL of 80% ethanol to the tube while on the magnetic rack and incubate at room temperature (∼20°C) for 30 s.
    vi. After the second wash, briefly spin the tube, return it to the magnetic rack.
    vii. Remove any residual ethanol and air dry the beads.
      **CRITICAL:** Avoid excessive drying of the beads, as this reduces recovery efficiency. Elute the samples when the beads appear dark brown and glossy.
    viii. Elute the ready to sequence BdLT-Seq barcode library DNA by adding 23 μL of TE buffer (NEB, cat. #E7760) and mix thoroughly by pipetting up and down at least 10 times.
    ix. Then incubate for 2 min at room temperature (∼20°C).
    x. Place the tube back onto the magnetic rack and transfer 20 μL of the supernatant into a fresh Eppendorf tube.
      **Pause point:** Store the library at −20°C or −80°C.
16. Determine BdLT-Seq barcode library concentration by using Qubit fluorometric quantification and further assess library’s quality by using Fragment Analyzer 5200 (Agilent, cat. #M5310AA) or alternative instruments (such as a Bioanalyzer).
17. Sequence the library using a MiSeq v2 Micro (Illumina, cat. # MS-103-1002; 4 million reads, with 15% PhiX) or alternative sequencing platforms (e.g., NovaSeq 6000, HiSeq 4000, NextSeq 2000).

### Cell transfection and preparation for BdLT-Seq experiment

**Timing: Highly dependent on the experimental window/requirements for the lineage tracing. BdLT-Seq can be performed as soon as 3 days after controlled plating or up to 30 days (experimentally verified).**

In this step, we provide a comprehensive protocol detailing the barcode-decay lineage tracing experimental setup. Our protocol encompasses the transfection of the barcoded episomes into the cells of interest and fluorescence-activated cell sorting of episome-containing cells, and provides recommendations to identify the optimal time window to conduct lineage tracing experiments.

18. Transfect approximately 1.2 million cells of interest with the BdLT-Seq episome library.
  a. To do this, plate 100,000 cells (adjustable based on cell type) per well in a six-well plate (9.6 cm^2^ per well) in 2 mL of cell culture medium.
  b. After 24 h, perform the transfection using Lipofectamine LTX reagent (Invitrogen, cat. #153385000).
    i. First, prepare Mix 1 in a fresh tube as follows:

**Table:** 

Reagent	Amount/well
Opti-MEM	180 μL
Plus Reagent	3 μL
    ii. Mix thoroughly by pipetting up and down 10 times.
    iii. Incubate for 15 min at room temperature (∼20°C).
    iv. Add 250 ng of BdLT-Seq barcoded episomes library generated in the previous steps and mix well by pipetting up and down 10 times.
    v. Incubate for 20 min at room temperature (∼20°C).
    vi. In parallel, prepare Mix 2 in a fresh tube as follows:

**Table:** 

Reagent	Amount/well
Opti-MEM	180 μL
Lipofectamine LTX reagent	7.2 μL
    vii. Thoroughly mix by pipetting up and down 10 times and incubate for 20 min at room temperature (∼20°C).
    viii. Next, combine Mix 1 and Mix 2 and mix well by pipetting up and down 10 times.
    ix. Incubate for an additional 20 min at room temperature (∼20°C).
    x. Add 360 μL of the combined mix per well to the cells in culture and mix well by gently swirling the plate.
    xi. Incubate for 24 h in a cell culture incubator (37°C, 5% CO_2_).
      ***Note:*** Alternative transfection reagents may be used to introduce the BdLT-Seq episome library into the chosen cell system.
  c. Twenty-four hours after transfection, carefully remove the medium and replace it with 2 mL of fresh pre-warmed medium.
  d. Incubate overnight (24 h) in a cell culture incubator (37°C, 5% CO_2_).
19. Forty-eight hours after transfection, collect cells from a single well and conduct flow cytometry analysis using the BD LSRFortessa X-20 Cell Analyzer to determine the efficiency of transfection (GFP-positive cells) using FlowJo software (BD, version 10.8.0).

***Note:*** We recommend gating on all cells using FSC-A/SSC-A, followed by doublet discrimination to include only single cells using FSC-A/FSC-H and finally gating on GFP-positive cells.

**Figure 2:**
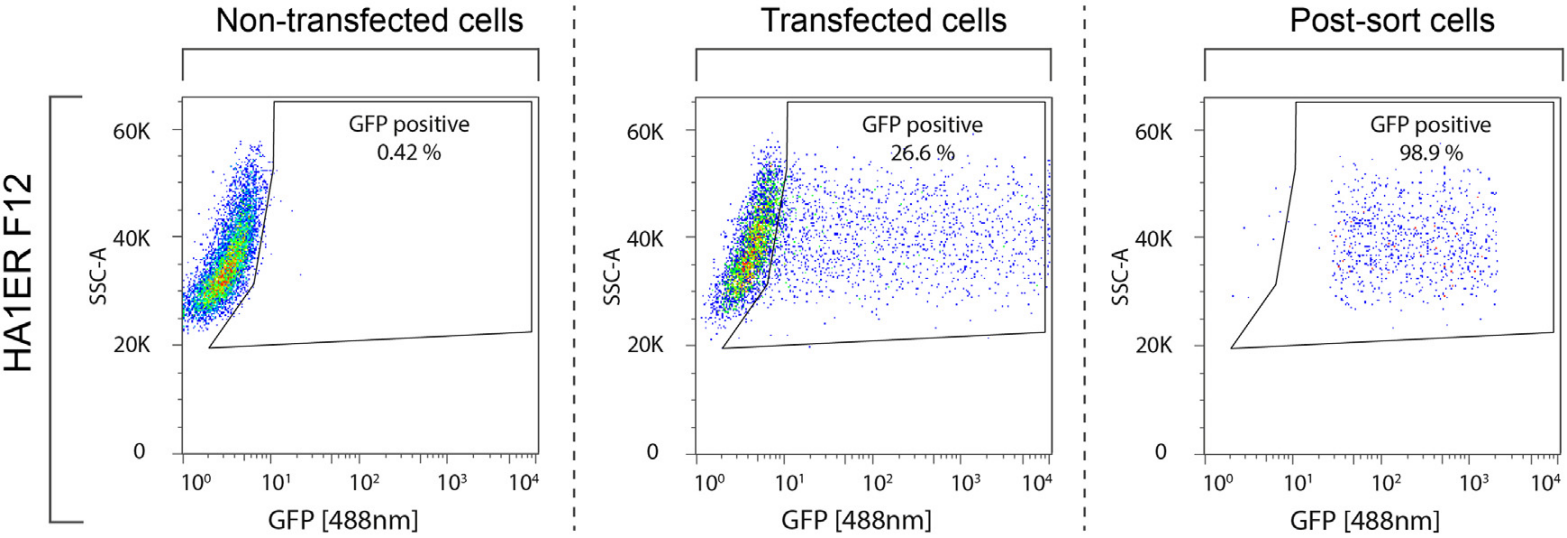
Experimental set-up for BdLT-Seq Scatter plots displaying data from flow cytometry assays assessing the percentage of H2B-GFP positive cells in non-transfected cells and cells transfected with the BdLT-Seq episome library pre- and post-FACS sorting of H2B-GFP positive cells in HA1ER cells (clone F12). Scatter plots from representative experiments are shown. Percentage of H2B-GFP positive cells is indicated.

20. If the number of GFP-positive cells is satisfactory (we aim to obtain at least 25% in our model systems), harvest the rest of the wells and proceed to collect GFP positive cells by fluorescence activated cell sorting (FACS) using either a BD FACSAria III Cell Sorter (BD Biosciences) or a BD InFlux Cell Sorter (BD Biosciences).
  a. Sort at least 50,000 cells.
  b. Perform an enrichment/purity assessment on the sorted sample (Figure 2).

**CRITICAL:** A post-sort enrichment/purity assessment must be conducted. Proceed with the experiment only if purity of GFP positive cells is 90% or higher, otherwise, untransfected or non-barcode expressing cells will be represented in the final sequencing library thus reducing the number of traceable lineages.

**CRITICAL:** Optimal transfection conditions may need to be established for a specific cell line of interest. To optimize transfection efficiency, we suggest end-users to test various amounts of Plus reagent and Lipofectamine LTX reagent. Alternatively, consider using different transfection reagents (e.g. Lipofectamine 2000, etc.) or alternative transfection methods (e.g. nucleofection).

***Note:*** Alternative flow cytometric analyzers and cell sorters capable of detecting GFP fluorescent signal can be used.

21. Sorted cells must be plated and allowed to proliferate under standard culture conditions for five to seven days (considering a cell replication rate of approximately 18–20 h).

**CRITICAL:** The replication rate for each evaluated cell line must be established prior to BdLT-Seq setup as it is crucial to determine sampling timings.

***Note:*** During the initial five days post sorting a barcode decay phase occurs, during which episomes are lost due to cell division rather than being inherited by the progeny (Phase I). Consequently, we recommend refraining from conducting any lineage tracing determination within this timeframe. The second phase of barcode decay (Phase II) begins around 5–7 days after transfection, during which the episomes present within cells stabilize while still exhibiting a characteristic episomal loss. This is the optimal time window to perform BdLT-Seq experiments. Note that this timeframe needs to be adjusted based on the proliferation rate of the cell system used. Thus, we suggest to adapt the timeframe accordingly, particularly if applying BdLT-Seq to cell lines that display significantly different proliferation rates.

22. Prepare and plate cells for lineage tracing.
  a. On the fifth to seventh day post-sorting, plate the cells to start the lineage tracing period.
    i. Trypsinize (Invitrogen, cat. #25300104) the cells and collect them via centrifugation.
    ii. Plate them at a density of 2,000 to 3,000 cells per well (96 well plate, 0.32 cm^2^, in 200 μL of cell culture medium).
  b. Allow the cells to proliferate for 5–7 days (or until ∼80,000 cells are reached, transfer to 24 well plate when necessary to avoid confluency).
  c. Following this, split the cells into two separate samples:
    i. Collect cells by trypsinization followed by centrifugation and resuspend in cell culture medium.
    ii. Divide the obtained cell suspension into two separate samples: sample T0 (Day 0) to be stored in liquid nitrogen (90% FCS, 10% DMSO), and sample T5/T7 (Day 5 or Day 7) where cells will be replated and will continue to expand in standard culture conditions for an additional five or seven days, depending on the experimental design.
  d. After five or seven days, split the cells again into two separate samples:
    i. Collect cells by trypsinization and centrifugation and resuspend in cell culture medium.
    ii. Again, divide the obtained cell suspension into two samples: sample T5/T7 (Day 5/Day 7) to be stored in liquid nitrogen (90% FCS, 10% DMSO), and sample T14 (Day 14) where cells will be replated and will expand in standard culture conditions for additional nine or seven days prior to being collected by trypsinization and stored in liquid nitrogen (90% FCS, 10% DMSO).
  e. The day before performing BdLT-Seq coupled to single-cell RNA sequencing, thaw all cryopreserved samples and return the cells to standard culture conditions for 24 h.

**CRITICAL:** The cell culture medium needs to be replaced (fresh and pre-warmed) as soon as the cells attach (approximately 8–10 h post-thawing) to remove the DMSO present in the cryopreservation media.

***Note:*** Experimental sampling time may be adapted following the experimental conditions to be evaluated. We have performed BdLT-Seq successfully for up to 30 days sampling every 10 days. We have also tested successfully shorter times down to 3 days.

***Note:*** If required, the number of sampling points may be adapted to include additional time points following a more complex experimental setup.

**CRITICAL:** The number of cells plated post-sorting is crucial for capturing and tracing the maximum number of lineages. As a starting point, we recommend to plate the exact number of cells that will be ultimately used for the scRNA-seq experiment and per condition/time point if multiplexing is required.

**Table:** 

Starting number of cells	Number of cells per sample included in scRNA-seq experiment
Max 2000 cells	2000 cells
Max 5000 cells	5000 cells

**CRITICAL:** All samples to be analyzed (BdLT-Seq + Gene Expression (GEx)) must be processed through a single 10× run to ensure proper side-by-side comparison avoiding altogether mathematical integration of the datasets. This can be easily achieved by balancing the number of required cells between all HTO-labeled samples (∼20,000) and split into individual 10× channels. If required, multiple 10× channels can be run in parallel to increase the number of cells to be analyzed. Immediately after the 10× run (post GEM-RT cleanup), ensure that all samples are pooled together to avoid the introduction of molecular biology bias throughout the processing of BdLT-seq/GEx samples.

### BdLT-seq: 10× Chromium processing, capture of BdLT-Seq barcoded mRNA and preparation of sequencing libraries

**Timing: 5–7 days, plus sequencing processing time**

In this step, we provide a step-by-step guide for generating the BdLT-seq sequencing library together with the scRNA-seq GEx library using the 10× Chromium platform.

23. Prepare cells for scRNA-seq.
  a. Prepare ‘HTO blocking buffer’ (5% FCS v/v in 1× PBS) and ‘HTO resuspension buffer’ (0.04% BSA w/v in 1× PBS). Filter-sterilize both before use and keep on ice.
  b. Collect cells by trypsinization followed by centrifugation, and resuspend them in 100–200 μL of HTO blocking buffer. Moving forward, keep cell suspensions on ice at all times.
  c. Wash the cells once in HTO blocking buffer.
  d. Determine the cell number using a Neubauer counting chamber (Marienfeld, cat. #0640030) or an alternative method (e.g., an automated cell counting device).
  e. Transfer approximately 50,000 to 100,000 cells into a fresh Eppendorf tube.
    ***Note:*** If the cell suspension is too diluted as evidenced by a low number of cells per ml, transfer the required number of cells into a fresh tube, spin it down and then resuspend the cells in 50 μL of HTO blocking buffer to reach ∼1000–2000 cells per μL.
    **CRITICAL:** Several steps in the experiment involve cell counting. Ensure that single cells are easily distinguishable and that no clumps or debris are present. If cell clumps are readily observed, filter the cell suspension using a cell strainer of suitable pore size. Filtering may lead to a reduction in cell numbers.
    **CRITICAL:** Please note that if multiplexing with HTO antibodies is performed, several washing steps will be required which could lead to the loss of an important number of cells. We recommend testing this aspect of the protocol to ensure that a sufficient number of cells are available prior to moving onto the next step (loading Chromium controller).
  f. Optional step: Use Hashtag antibodies coupled to specific oligonucleotides (HTO) (BioLegend, cat. #A0251, A0252, A0253, A0254, A0255, A0256, A0257, A0258, A0259, A0260, clone LNH-94 2M2, 0.5 mg/mL) to label individual samples for multiplexing.
    i. Add 1 μL of stock HTO antibodies to each sample of 50 μL, to reach a final concentration of 10 μg/mL and a ratio of 0.5 μg per 50,000 cells.
    ii. Mix thoroughly and incubate for 30 min on ice.
  g. Wash cells three times using HTO resuspension buffer.
  h. After the last wash, resuspend the cells in 50 μL HTO resuspension buffer.
  i. Immediately after, count cells using Neubauer counting chamber (or alternative approaches) using trypan blue (stock 0.4% w/v; dilute 1:1 with cells) to assess the total number of retrieved cells and to assess the percentage of dead cells (trypan blue positive).
    ***Note:*** Ensure high viability of the cells (∼4% trypan blue positive cells or less).
  j. Only shortly before the Chromium run, combine HTO labeled samples into a single sample based on the experimental conditions and load 20,000 cells (for the expected outcome of 10,000 cells) onto the 10× Chromium controller (10× Genomics, cat. #1000204).
    ***Note:*** Optimal number of cells to be loaded onto the Chromium controller ensuring optimal performance may need to be determined (minimum number of cells loaded into the controller for maximum number of single cells retrieved). Please bear in mind that the origin/nature of the sample plays a significant role at this step. We recommend conducting tests to ensure optimal performance of BdLT-Seq.
24. Process the sample following the standard 10× Chromium protocol (Chromium Next GEM Single Cell 3ʹ Kit v3.1, 10× Genomics, cat. #1000269) up until the cDNA amplification step (steps 1.0–2.1). For the detailed protocol, refer to the Chromium Next GEM Single Cell 3ʹ Kit v3.1 manual. Briefly, the following steps need to be performed:
  a. Load the Chromium NextGEM Chip G and run the Chromium Controller.
  b. After completion of the run, transfer the GEMs and start the incubation for the GEM-RT reaction.
  c. Perform the post GEM-RT cleanup using Dynabeads MyOne SILANE and transfer the eluted sample (35 μL) to a new PCR tube.
25. Continue with the cDNA amplification step (Step 2.2 in the 10× Chromium protocol, version 3.1).
  a. If using HTOs, supplement the cDNA amplification reaction with the HTO additive primer (0.2 μM stock solution freshly prepared just before use).
  b. Prepare the cDNA amplification mix on ice:

**Table:** 

Reagent	Amount/reaction
Amp Mix	50 μL
cDNA primers	15 μL
HTO_additive (0.2 μM)	1 μL
  c. Vortex the mix and spin down briefly before adding 65 μL of the cDNA amplification mix to the 35 μL of sample.
  d. Pipette mix 15 times, spin down briefly and begin the cDNA amplification in a thermal cycler using the following protocol:

**Table:** 

Step	Temperature	Time	Cycles
Initial Denaturation	98°C	3 min	1
Denaturation	98°C	15 s	11–13 cycles
Annealing	63°C	20 s	
Extension	72°C	1 min	
Final extension	72°C	1 min	1
Hold	4°C

    ***Note:*** The recommended starting point to optimize the number of cycles depends on the targeted number of cells to be recovered as determined when loading the Chromium controller: 13 cycles for <500 cells, 12 cycles for 500–6,000 cells and 11 cycles for >6,000 cells. The optimal cycle number may need to be determined depending on the cell line to be analyzed and/or the experimental conditions.
26. Perform cDNA clean-up to separate the HTO fraction from the cDNA (Figures 3A–3C) using SPRI beads (e.g., Beckman Coulter, AMPure XP, cat. #A63880) as outlined in Step 2.3 of the standard 10x Chromium protocol with feature barcoding technology for cell surface proteins (10× Genomics, version 3.1, cat. #1000269).
  a. Briefly, resuspend the SPRI beads by vortexing and add 60 μL (0.6×) to each sample.
  b. Mix by pipetting up and down 15 times and incubate for 5 min at room temperature (∼20°C).
  c. Place the tube on the magnetic rack.
  d. Once the solution looks clear, transfer 80 μL of the supernatant to a new Eppendorf tube. Keep the transferred supernatant at room temperature (∼20°C) and save it for the later clean-up of the HTO cDNA fraction.
  e. Remove the remaining supernatant from the pellet without disturbing it and immediately continue with the pellet clean-up for the gene expression cDNA fraction (GEx) as follows:
    i. Add 200 μL of 80% ethanol to the pellet and wait 30 s to remove the ethanol without disturbing the pellet by gentle pipetting.
    ii. Repeat this washing step once.
    iii. Briefly centrifuge the tube and place it back onto the magnetic rack.
    iv. Remove any remaining ethanol and air dry for a maximum of 2 min.
    v. Take the tube off the magnetic rack, add 40.5 μL Buffer EB and pipette mix 15 times.
    vi. Incubate for 2 min at room temperature (∼20°C) to elute.
    vii. Return the tube to the magnetic rack until the separation is complete.
    viii. Then transfer 40 μL of the GEx cDNA sample to a fresh Eppendorf tube.
  f. For the clean-up of the supernatant (HTO cDNA fraction) that was set aside after the SPRI bead-based separation, continue as follows:
    i. Vortex the SPRI bead reagent and add 70 μL of SPRI beads (2.0×) to the 80 μL of supernatant and mix well by pipetting up and down 15 times.
    ii. Incubate for 5 min at room temperature (∼20°C).
    iii. Place the tube on the magnetic rack and once the separation is complete, remove and discard the supernatant.
    iv. While on the magnetic rack, wash the pellet twice by adding 200 μL of 80% ethanol and incubating for 30 s.
    v. After the second wash, briefly spin down and return the tube to the magnetic rack to remove any remaining ethanol.
    vi. Air dry for a maximum of 2 mi.
    vii. Remove the tube from the magnet and add 40.5 μL Buffer EB.
    viii. Incubate for 2 min at room temperature (∼20°C) for elution.
    ix. Place the tube back onto the magnet and once separation is complete, transfer 40 μL supernatant containing the HTO cDNA fraction to a new Eppendorf tube.

**Figure 3:**
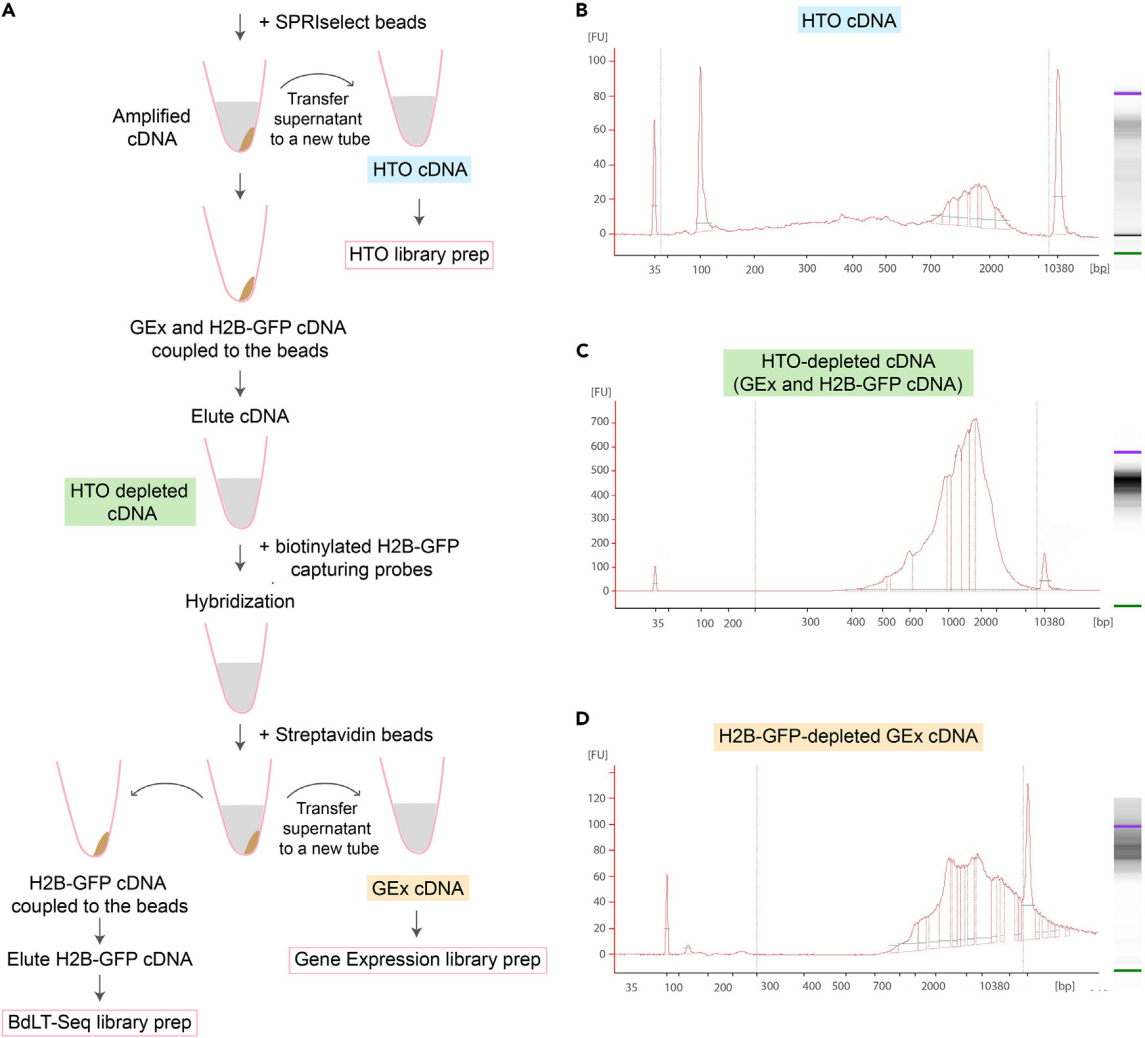
BdLT-Seq workflow and cDNA quality controls (A) Scheme representing the BdLT-Seq workflow within the 10× v3.1 experimental pipeline. Major steps for library construction are depicted as color coded sections. (B) Representative electropherogram (Bioanalyzer) of purified HTO cDNA fraction. (C) Representative electropherogram (Bioanalyzer) of purified HTO-depleted cDNA fraction. (D) Representative electropherogram (Bioanalyzer) of purified H2B-GFP-depleted Gene Expression (GEx) cDNA fraction.
27. Immediately after finishing clean-up of the supernatant (HTO cDNA), continue with the quality control analysis (QC) and use the GEx cDNA fraction to capture H2B-GFP cDNA (containing BdLT-Seq barcodes) (see subsequent steps below).
  a. Conduct a quality control analysis (QC) of the purified GEx and HTO fractions using the Fragment Analyzer 5200 (Agilent, cat. #M5310AA) or alternative instruments such as a Bioanalyzer (Figures 3B and 3C).

**Pause point:** Purified HTO cDNA sample can be stored at −20°C for up to 4 weeks before continuing with construction of the sequencing library.

**CRITICAL:** Immediately continue the protocol for the HTO-depleted cDNA fraction (GEx cDNA).

28. Concentrate the HTO-depleted cDNA fraction (GEx cDNA) fraction using Dynabeads MyOne Silane beads (Invitrogen, cat. #37002D).
  a. Wash 5 μL of beads twice with 500 μL of RLT lysis buffer (QIAGEN, cat. #79216) using a magnetic rack.
  b. Resuspend beads in 140 μL of RLT buffer (3.5× the initial sample volume) and add it to the 40 μL of the HTO-depleted cDNA fraction.
  c. Next, add 180 μL of absolute ethanol to the reaction (4.5× the initial sample volume) ensuring thorough mixing by pipetting at least 10 times.
  d. Incubate for 15 min at room temperature (∼20°C) using a ThermoMixer C (300 rpm, Eppendorf, cat. #EP5382000015).
  e. Place the reaction on a magnet and once the separation is complete, discard the supernatant and perform three washes with 80% ethanol.
  f. After the third wash, briefly spin the tube and return it to the magnetic rack.
  g. Remove any remaining ethanol and allow the beads to air dry for 1–2 min at room temperature (∼20°C).
    **CRITICAL:** Avoid excessive drying of the beads, as this could lead to reduced recovery efficiency. Elute the samples when the beads appear dark brown and glossy.
  h. Elute the material in 5 μL of nuclease-free water by incubation for 10–15 min at room temperature (∼20°C) in a ThermoMixer C at 300 rpm.
29. Utilize the concentrated HTO-depleted cDNA fraction (GEx cDNA) to capture H2B-GFP cDNA, which contains the BdLT-Seq barcodes (Figure 3A), using a set of four pooled strand-specific biotinylated probes for H2B-GFP mRNA.
  ***Note:*** We recommend purifying biotinylated probes (purchase as cartridge-RP1 purified) before attempting H2B-GFP capture. Purchased strand-specific probes should be size-separated on a PAGE-Urea gel and probes of the expected molecular weight must be retrieved from the gel and purified (see preparative step at the beginning of this protocol: ‘Preparation of strand-specific biotinylated probes directed towards H2B-GFP mRNA’). Probe purification must be done in a preparative manner. Probes can be stored at −20°C/−80°C.
  ***Note:*** Alternatively, PAGE-purified strand-specific probes can be directly ordered and used in the hybridization and capture reaction. However, we strongly recommend to PAGE-purify the probes in-house, to ensure the highest yield and quality.
  a. Hybridize the probes using xGen Hybridization and Wash Kit (IDT, cat. #1080577) following the manufacturer’s instructions:

**Table:** 

Reagent	Amount/tube
Gen 2× Hybridization buffer	8.5 μL
Gen Hybridization buffer enhancer	2.7 μL
HTO depleted cDNA fraction	5 μL
Probes (4 pmol/μL, pool of 4 probes)	2 μL

    i. Mix well by pipetting and incubate for 10 min at room temperature (∼20°C).
    ii. Proceed with the following thermocycler program: 30 s at 95°C, followed by 16 h at 65°C (lid temperature 105°C).
  b. The next day, prepare buffers according to the user manual for the xGen Hybridization and Wash Kit (IDT, cat. #1080577):

**Table:** 

Component	Nuclease-free water (μL)	Buffer (μL)	Total (μL)
xGen 2× Bead wash buffer	150	150	300
xGen 10× Wash Buffer 1	225	25	250
xGen 10× Wash Buffer 2	135	15	150
xGen 10× Wash Buffer 3	135	15	150
xGen 10× Stringent Wash Buffer	270	30	300

    ***Note:*** If xGen 10× Wash Buffer 1 appears cloudy, heat it to 65°C. Prepare aliquots of 100 μL of 1× Wash Buffer 1 and 300 μL of 1× Stringent Wash Buffer in separate tubes to pre-warm them in a thermomixer at 65°C.
  c. Prepare the Bead Resuspension Buffer:

**Table:** 

Reagent	Amount/reaction
Gen 2× Hybridization buffer	8.5 μL
Gen Hybridization buffer enhancer	2.7 μL
Nuclease-free water	5.8 μL
  d. Equilibrate Dynabeads M-270 Streptavidin (IDT, cat. #1080583) at room temperature (∼20°C) for 30 min.
  e. After hybridization, capture the H2B-GFP fraction using Dynabeads M-270 Streptavidin magnetic beads.
    i. First, ensure magnetic streptavidin beads are thoroughly mixed by vortexing before use.
    ii. Wash 20 μL of magnetic streptavidin beads twice with 100 μL of xGen 1× Bead Wash Buffer, place the mix on a magnetic rack and remove the supernatant.
    iii. Resuspend the beads in 17 μL of previously prepared Bead Resuspension Buffer.
    iv. Next, add the 17 μL of resuspended streptavidin beads to the hybridization reaction and mix thoroughly by pipetting up and down at least 10 times.
    v. Incubate the reaction in a thermocycler at 65°C for 45 min (with a lid temperature of 70°C), making sure to pipette-mix the reaction every 10 min.
  f. Washes and elution of captured H2B-GFP molecules.
    i. During the incubation of the capture reaction, preheat the aliquots of Wash Buffer 1 (100 μL) and Stringent Buffer (300 μL) in a thermomixer at 65°C. Ensure that Wash Buffer 1 and Stringent Buffer are kept in the thermomixer during incubations so buffers are constantly kept at 65°C.
    ii. After incubation of the capture reaction, add 100 μL Wash Buffer 1 pre-warmed to 65°C to the sample.
    iii. Gently mix the solution by pipetting it up and down 10 times, then place it on the magnet.
    iv. Once separation is complete, transfer the supernatant into a fresh tube and keep it on ice (this fraction will be used for the 10× gene expression library - GEx fraction).
    v. Wash the beads by adding 150 μL of Stringent Buffer warmed at 65°C and gently mix the solution by pipetting it up and down 10 times.
    vi. Incubate in a thermocycler for 5 min at 65°C (lid temperature 70°C).
    vii. Afterwards, place the suspension on a magnet and discard the supernatant once separation is complete.
    viii. Repeat the previous three steps to wash again with Stringent Buffer.
    ix. Add 150 μL of room temperature (∼20°C) Wash Buffer 1 to the beads and gently mix the solution by pipetting up and down 10 times.
    x. Incubate for 2 min at room temperature (∼20°C), then place the tube on a magnet and once separation is complete, discard the supernatant.
    xi. Add 150 μL of room temperature (∼20°C) Wash Buffer 2 to the beads and gently mix the solution by pipetting it up and down 10 times.
    xii. Incubate for 2 min at room temperature (∼20°C), then place the tube on a magnet and once separation is complete, discard the supernatant.
    xiii. Add 150 μL of room temperature (∼20°C) Wash Buffer 3 to the beads and gently mix the solution by pipetting it up and down 10 times.
    xiv. Incubate for 2 min at room temperature (∼20°C), then lace the tube on a magnet and once separation is complete, discard the supernatant.
    xv. To elute H2B-GFP cDNA harboring BdLT-Seq barcodes, add 100 μL of denaturation buffer (100 mM NaOH, 0.1 mM EDTA) to the bead-cDNA complex and incubate for 15 min at room temperature (∼20°C) in a ThermoMixer C (300 rpm).
    xvi. Place the tube on a magnetic rack. Once separation is complete, transfer the supernatant into a fresh tube.
    xvii. To stop the reaction, add 10 μL of 1 M Tris buffer (pH 7.5) and 10 μL of 1 M HCl.
30. Purify the H2B-GFP cDNA fraction (BdLT-Seq fraction) as well as the GEx fraction using Dynabeads MyOne Silane beads (Invitrogen, cat. #37002D).
  a. To do this, pipette 10 μL (for 2 samples) of Dynabeads MyOne Silane beads (Invitrogen, cat. #37002D) and wash the beads twice in 500 μL of RLT lysis buffer (QIAGEN, cat. #79216).
  b. Next, resuspend the beads in 840 μL of RLT buffer (3.5× initial sample volume) and add 420 μL to the ∼120 μL of each sample.
  c. Add 540 μL of absolute ethanol to each reaction (4.5× initial sample volume) and mix well by pipetting.
  d. Incubate for 15 min at room temperature (∼20°C) in a ThermoMixer C (300 rpm).
  e. Place the samples on a magnetic rack and once the separation is complete, discard the supernatant.
  f. Wash the beads three times with 500 μL of 80% ethanol.
  g. After the last wash, briefly spin down the tube, put it back onto the magnetic rack, remove the residual ethanol and air dry the beads for 1–2 min at room temperature (∼20°C).
    **CRITICAL:** Avoid excessive drying of the beads prior to the elution step, as this could lead to reduced recovery efficiency. Elute the samples when beads appear dark brown and glossy.
  h. Elute the H2B-GFP BdLT-Seq barcoded fraction by adding 20 μL of nuclease-free water and elute the GEx cDNA fraction by adding 40 μL of EB buffer.
  i. Incubate both samples for 10–15 min at room temperature (∼20°C) in a ThermoMixer C at 300 rpm.
31. Perform quality control of GEx cDNA fraction using Fragment Analyzer 5200 (Agilent, cat. #M5310AA) or alternative instruments such as a Bioanalyzer (Figure 3D).

***Note:*** The size of the GEx cDNA fraction may exhibit a slight shift towards higher molecular weight (Figures 3A and 3C). This is a normal occurrence and does not impact library construction or final sequencing output.

**Pause point:** Store GEx cDNA sample at −20°C for up to 4 weeks or continue straight away with sequencing library preparation.

32. Use 120 ng of the GEx cDNA fraction to generate the 10× gene expression library following the 10x Chromium protocol (fragmentation, end repair, A-tailing and library construction steps. 10× Genomics, version 3.1, cat. #1000269)
  a. Fragmentation, end repair and A-tailing reaction.
    i. Prepare a thermocycler with the following incubation protocol and pre-cool it to 4°C:

**Table:** 

Steps	Temperature	Time
Lid temperature	65°C	
Pre-cooling	4°C	hold
Fragmentation	32°C	5 min
End Repair and A-tailing	65°C	30 min
Hold	4°C
    ii. Vortex to mix the fragmentation buffer and verify no precipitate is present.
    iii. Prepare the fragmentation mix on ice as specified below, pipette mix 10 times and centrifuge briefly:

**Table:** 

Reagent	Amount / reaction
Fragmentation buffer	5 μL
Fragmentation enzyme	10 μL
    iv. Transfer 120 ng of purified GEx cDNA to a PCR tube and add Buffer EB to a final volume of 35 μL.
    v. Add 15 μL of the fragmentation mix, mix well by pipetting 15 times and spin down briefly.
    vi. Place the tube into the pre-cooled thermocycler and start the pre-programmed protocol by skipping the initial 4°C holding step.
      ***Note:*** The remaining GEx cDNA sample can be stored at −20°C for up to 4 weeks to generate additional 3′ gene expression libraries if required.
  b. Following fragmentation, end repair and A-tailing, purify the required molecules by double-sided size-selection using SPRI beads.
    i. For this, vortex the SPRI beads well to mix and add 30 μL of SPRI beads (0.6×) to each sample (50 μL).
    ii. Mix well by pipetting 15 times and incubate at room temperature (∼20°C) for 5 min.
    iii. Place the tube onto the magnetic rack and wait until the solution clears.
    iv. Transfer 75 μL of the supernatant to a new Eppendorf tube and discard the previous tube with the pelleted beads.
    v. After vortexing the SPRI beads, add 10 μL of SPRI beads to the 75 μL of transferred supernatant.
    vi. Pipette mix 15 times and incubate for 5 min at room temperature (∼20°C).
    vii. Separate using the magnetic rack and once separation is complete, remove and discard 80 μL of supernatant.
    viii. Keep the beads on the magnetic rack and add 125 μL of 80% ethanol to wash and remove after 30 s.
    ix. Repeat this washing step once more for a total of two washes.
    x. Centrifuge briefly, place tube back onto the magnet and remove any remaining ethanol.
    xi. Air dry for 1 min, make sure not to over dry the beads for maximum elution efficiency.
    xii. Remove from the magnet, add 50.5 μL Buffer EB and mix by pipetting 15 times.
    xiii. Incubate for 2 min at room temperature (∼20°C to elute.
    xiv. Place on the magnet and once separation is complete, transfer 50 μL to a fresh Eppendorf tube.
  c. Following purification, perform adapter ligation.
    i. Prepare the following adaptor ligation mix, pipette mix 15 times and centrifuge briefly:

**Table:** 

Reagent	Amount / reaction
Ligation buffer	20 μL
DNA ligase	10 μL
Adaptor oligos	20 μL
    ii. Add 50 μL of the prepared adaptor ligation mix to 50 μL sample, pipette mix 15 times and centrifuge briefly.
    iii. Use the following program to incubate in a thermocycler:

**Table:** 

Steps	Temperature	Time
Lid temperature	30°C	
Adaptor ligation	20°C	15 min
Hold	4°C
  d. Perform a post-ligation clean-up of the sample.
    i. For this, vortex SPRI beads to resuspend them and add 80 μL (0.8×) of SPRI beads to 100 μL of sample.
    ii. Pipette 15 times to mix and incubate for 5 min at room temperature (∼20°C).
    iii. Place on the magnet and once separation is complete, remove and discard the supernatant.
    iv. Leaving the tube on the magnet, wash by adding 200 μL 80% ethanol and removing after 30 s.
    v. Repeat this washing step once more for a total of two washes.
    vi. After the second wash, briefly centrifuge the tube, place back onto the magnet and remove any remaining ethanol.
    vii. Air dry for a maximum of 2 min, making sure not to over-dry the beads.
    viii. Remove from the magnet, add 30.5 μL Buffer EB and mix by pipetting 15 times.
    ix. Incubate for 2 min at room temperature to elute (∼20°C).
    x. Place onto the magnet and once separation is complete, transfer 30 μL to a fresh Eppendorf tube.
  e. For the final sample index PCR for sequencing library construction, make sure to choose appropriate sample index primers so that no sample indices overlap in a multiplexed sequencing run and record which sample indices were used.
    i. Add 50 μL of Amp Mix to 30 μL sample and add 20 μL of an individual Dual Index TT Set A (cat. #1000215) well.
    ii. Incubate in a thermocycler using the following PCR protocol using 11 cycles:

**Table:** 

Steps	Temperature	Time	Cycles
Lid temperature	105°C		
Initial Denaturation	98°C	45 s	1
Denaturation	98°C	20 s	11 cycles
Annealing	54°C	30 s	
Extension	72°C	20 s	
Final extension	72°C	1 min	1
Hold	4°C

      ***Note:*** PCR cycles may need to be optimized on a case-to case basis. We recommend using 11 cycles as starting point for optimization.
  f. Following sample index PCR, purify the final sequencing library using SPRI beads.
    i. To do so, vortex the SPRI beads to resuspend and add 60 μL of SPRI beads (0.6×) to 100 μL of sample.
    ii. Pipette mix 15 times and incubate for 5 min at room temperature (∼20°C).
    iii. Place on the magnet and once separation is complete, transfer 150 μL of the supernatant to a new tube and discard the previous tube containing pelleted beads.
    iv. Vortex the SPRI beads and add 20 μL of SPRI beads (0.8×) to the transferred supernatant.
    v. Mix well by pipetting 15 times and incubate for 5 min at room temperature (∼20°C).
    vi. Place the tube on the magnet and upon completion of the separation, remove and discard 165 μL of supernatant without disturbing the beads.
    vii. Keep the tube on the magnet and add 200 μL of 80% ethanol incubating for 30 s to wash the beads.
    viii. Repeat this washing step once more for a total of two washes.
    ix. After the second wash, centrifuge briefly, place back onto the magnet and remove any remaining ethanol.
    x. Remove the tube from the magnet and add 35.5 μL Buffer EB and mix by pipetting 15 times.
    xi. Incubate for 2 min at room temperature (∼20°C) to elute.
    xii. Place tube back onto the magnetic rack and once separation is complete, transfer 35 μL of the final GEx sequencing library sample to a new tube.
      **Pause point:** Store the GEx library at −20°C or −80°C.
  g. Quantify the obtained GEx library DNA using Qubit and assess the library quality on a Fragment analyzer 200 (Agilent, cat. #M5310AA; Figure 4A) or alternative instruments such as a Bioanalyzer.
33. Perform a PCR amplification to generate the HTO library by amplifying the HTO cDNA using primers specifically targeting the HTO on the 5′-end and Read 1 on the 3′-end. Given the primer design, this step generates standard Illumina paired-end sequencing constructs bearing P5 and P7 anchoring sequences.
  a. Prepare the PCR mix for the HTO library generation as follows:

**Table:** 

Reagent	Amount
DNA template – HTO cDNA	5 μL
Expand High Fidelity Enzyme Mix	0.75 μL
Forward primer: HTO_library_Forward primer_BC1/2/3 (20 μM)	1 μL
Reverse primer: HTO_library_Reverse primer (20 μM)	1 μL
dNTPs (10 mM each)	1 μL
Expand High Fidelity Buffer (10×) with 15 mM MgCl_2_	5 μL
Nuclease-free water	36.25 μL
  b. Mix well and perform the PCR amplification using the following conditions:

**Table:** 

Steps	Temperature	Time	Cycles
Initial Denaturation	94°C	2 min	1
Denaturation	94°C	15 s	14–16 cycles
Annealing	64°C	30 s	
Extension	72°C	30 s (+5 s in every cycle starting from cycle 11)	
Final extension	72°C	7 min	1
Hold	4°C
  c. Purify the HTO library using SPRI beads.
    i. To do this, add 60 μL (1.2×) of resuspended SPRI beads to the PCR reaction.
    ii. Thoroughly mix by pipetting at least 10 times and incubate for 5 min at room temperature (∼20°C).
    iii. Next, place the tube on a magnet to separate the beads from the solution.
    iv. After the solution becomes clear, carefully remove the supernatant.
    v. Wash the beads twice by adding 200 μL of 80% ethanol to the tube while on the magnetic rack and incubate for 30 s at room temperature (∼20°C) after each wash.
    vi. Following the second wash, give the tube a brief spin, return it to the magnetic rack.
    vii. Remove any residual ethanol and let the beads air dry.
      **CRITICAL:** Avoid over-drying the beads, as this could reduce recovery efficiency. Elute samples when beads appear dark brown and glossy.
    viii. Elute the HTO library by adding 40 μL of nuclease-free water and mixing thoroughly by pipetting up and down at least 10 times.
    ix. Incubate for 2 min at room temperature (∼20°C) to elute.
    x. Place the tube back on the magnetic rack and transfer 40 μL of the supernatant into a fresh tube.
      **Pause point:** Store the HTO library at −20°C or −80°C.
  d. Quantify the amount of extracted HTO library DNA using Qubit fluorometric quantification and assess the library quality using a Fragment Analyzer or an alternative instrument such as a Bioanalyzer (Figure 4B).
    ***Note:*** The TSO-RT-oligo product (∼150 bp) can be amplified during the HTO PCR by carryover primers from the cDNA amplification reaction. This PCR product will not cluster during sequencing but will interfere with library quantification. To further enrich the HTO specific product, re-do the PCR amplification by adding 1–2 additional cycles and assess the library quality using a Fragment Analyzer or an alternative instrument. Alternatively, additional SPRI beads purification can be performed after cDNA amplification to reduce primer carryover.
34. The BdLT-Seq sequencing library is generated by amplifying the H2B-GFP fragment (cDNA) that contains the BdLT-Seq barcodes using primers that specifically target H2B-GFP cDNA on the 5′-end and Read 1 on the 3′-end. This step generates standard Illumina paired-end sequencing constructs including the P5 and P7 anchoring sequences.
  a. Perform a PCR amplification to generate the BdLT-Seq sequencing library:

**Table:** 

Reagent	Amount
DNA template – H2B-GFP cDNA	20 μL
Expand High Fidelity Enzyme Mix	0.75 μL
Forward primer: LT_H2B-GFP_library_Forward primer_BC1/2/3 (20 μM)	1 μL
Reverse primer: LT_H2B-GFP_library_Reverse primer (20 μM)	1 μL
dNTPs (10 mM each)	1 μL
Expand High Fidelity Buffer (10×) with 15 mM MgCl_2_	5 μL
Nuclease-free water	21.25 μL
  b. Mix well and perform the BdLT-Seq PCR amplification as follows:

**Table:** 

Steps	Temperature	Time	Cycles
Initial Denaturation	94°C	2 min	1
Denaturation	94°C	15 s	21 cycles
Annealing	64°C	30 s	
Extension	72°C	45 s (+5 s in every cycle starting from cycle 11)	
Final extension	72°C	7 min	1
Hold	4°C
  c. Perform agarose gel electrophoresis to purify the BdLT-Seq library.
    i. To do so, prepare 300 mL of a 1.4% agarose solution (w/v) in 1× TAE buffer.
    ii. After melting the agarose in a microwave, allow it to cool to ∼60°C.
    iii. Add 3 μL of Ethidium Bromide (10 mg/mL) and mix thoroughly by swirling.
    iv. Pour the mixture into a gel tray, insert a suitable comb (estimate 45 μL per well) and let the gel set.
      **CRITICAL:** Ethidium Bromide is a mutagen and carcinogen. Therefore, cautious handling is necessary when dealing with gels containing this compound. Always wear gloves when working with Ethidium Bromide solution or gels that contain it. Opt for Ethidium Bromide solution instead of the powder form or use an alternative DNA dye such as SYBR Safe or Gel Red. Please use a plastic wrap when positioning the gel in the UV apparatus to avoid potential contamination.
    v. Combine 50 μL of the BdLT-Seq PCR reaction with 10 μL of 6× DNA loading dye.
    vi. After the gel has set, load 40–50 μL of the mixture into each well.
    vii. Include a well containing a double-stranded DNA molecular weight ladder (100 bp).
      **CRITICAL:** Ensure that empty wells are left between different samples and between samples and the DNA molecular weight ladder. This precaution is essential to prevent cross-contamination between wells.
    viii. Conduct gel electrophoresis at 150 V in 1× TAE buffer until the lower dye (bromophenol blue) migrates to approximately 5 cm from the bottom of the gel (20 cm gel).
    ix. After switching off the current, take the gel out of the tray, lay it on a sheet of plastic wrap, and expose it to UV illumination from a transilluminator at low lamp power.
      ***Note:*** Ensure that gel electrophoresis is performed for a sufficient duration to obtain a proper separation of the DNA fragments analyzed. Failure to do so might compromise the purified material.
      ***Note:*** Ethidium Bromide is positively charged and migrates towards the cathode, while the DNA molecules migrate towards the anode. As gel electrophoresis proceeds, Ethidium Bromide gradually moves out of the gel, resulting in reduced fluorescent intensity towards the gel's end.
    x. Excise a gel slice of approximately 3–4 mm in size, corresponding to the expected molecular weight (expected size: 410 bp).
    xi. Use the MinElute Gel Extraction Kit (QIAGEN, cat. #28604) to purify the BdLT-Seq library from the gel following the manufacturer’s instructions and elute in 20 μL of nuclease-free water.
      **Pause point:** Store the BdLT-Seq library at −20°C or −80°C.
  d. Quantify the amount of extracted DNA using Qubit and assess the quality of the BdLT-Seq library using a Fragment Analyzer or an alternative instrument such as a Bioanalyzer (Figure 4C).
35. Sequence the Gene Expression (GEx), HTO and the Lineage Tracing (BdLT-Seq) libraries on a NovaSeq 6000 version 1.5 apparatus (Illumina, cat. #20013850).
  a. Required Sequencing depth:
    i. GEx library – 40,000–50,000 reads per cell.
    ii. HTO library – 2,000–3,000 read pairs per cell.
    iii. GFP library –10,000–12,000 read pairs per cell.
  b. Sequencing type: paired-end, dual indexing.
  c. Sequencing read: Read 1–28 cycles, i7 Index – 10 cycles, i5 Index – 10 cycles, Read 2–90 cycles.

**Figure 4:**
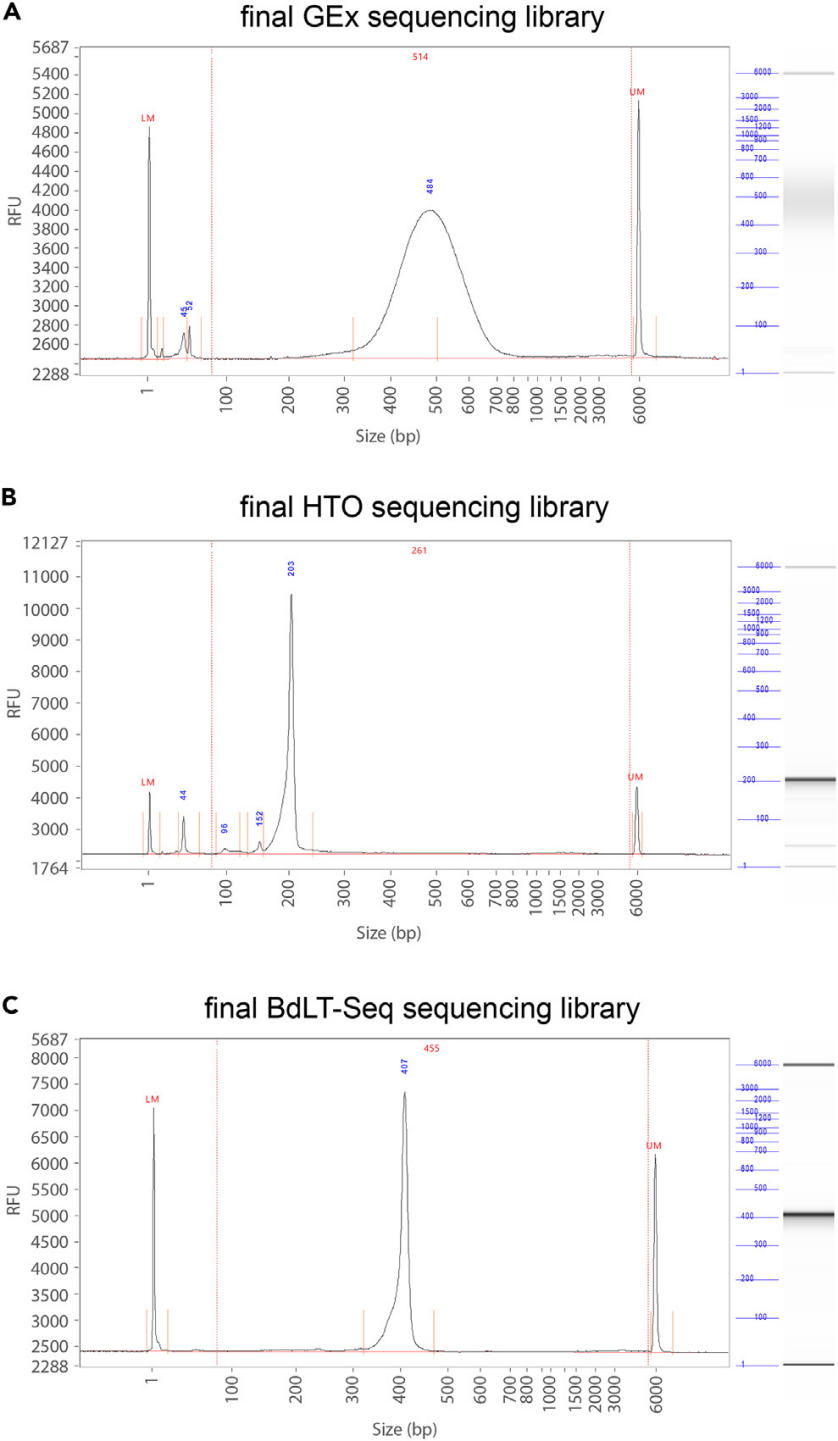
Sequencing libraries (BdLT-Seq, HTO and Gene Expression) quality controls (A) Representative electropherogram (Agilent Fragment Analyzer) of the final Gene Expression sequencing library (GEx). (B) Representative electropherogram (Agilent Fragment Analyzer) of the final HTO library. (C) Representative electropherogram (Agilent Fragment Analyzer) of the final BdLT-Seq library.

***Note:*** Alternative sequencing platforms such as NextSeq (Illumina) can be used.

## Expected outcomes

The BdLT-Seq protocol allows to generate a complex library of barcoded episomes that can be utilized for directional lineage tracing experiments and which is fully compatible with state-of-the-art single-cell RNA sequencing platforms (e.g., Chromium 10× Genomics). Notably, our methodology enables the tracking of transcriptome transitions and transcriptome inheritance, as well as the relationships among divergent molecular states in a directional and time-resolved manner. Thus, the application of BdLT-Seq enables the construction of directional lineage trees, opening a path to study cell plasticity dynamics while preserving isogeneity. Due to its universal experimental setting, BdLT-Seq can be applied to multiple cellular systems and experimental conditions such as basal growth and in response to various environmental cues.^1^

## Quantification and statistical analysis

**Figure 5:**
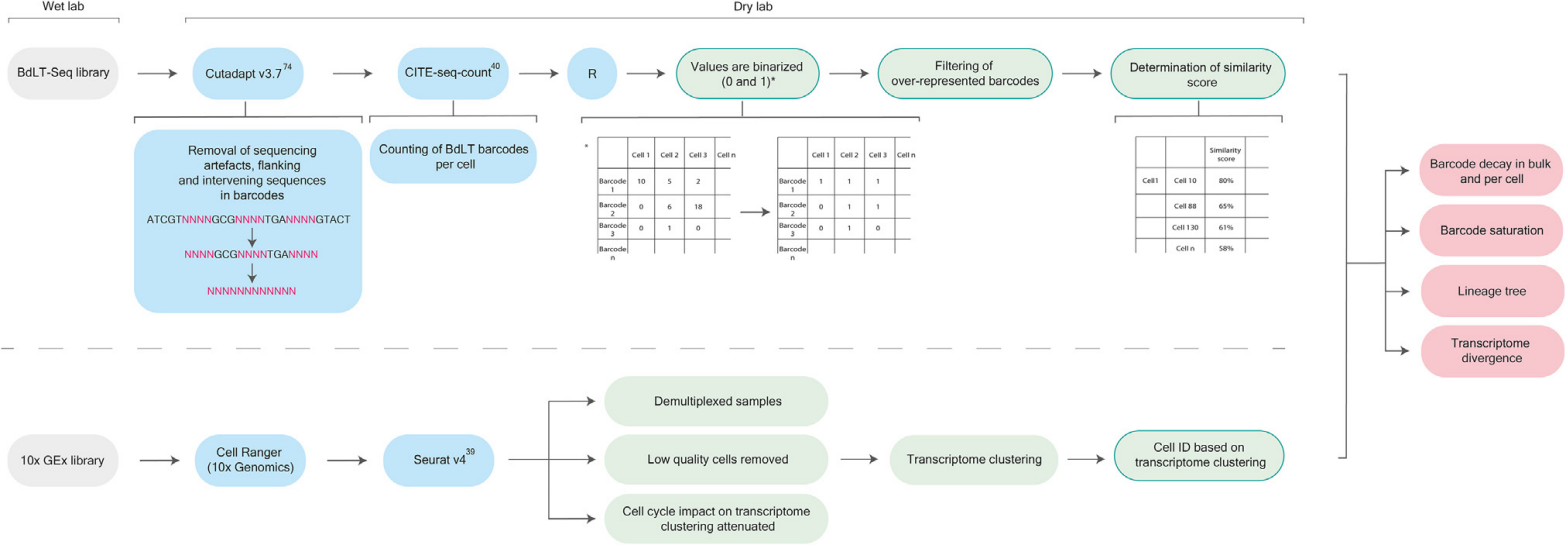
Computational pipeline for BdLT-Seq da-ta analysis Scheme representing computational pipeline used for BdLT-Seq analysis. Two independent libraries – one for gene expression (10× GEx library) and the other one for lineage tracing (BdLT-Seq library) are prepared. Independent computational pipelines are applied for data analysis and combined as depicted.

BdLT-Seq data analysis is straight forward and takes advantage of state-of-the-art benchmarked computational pipelines. Typical BdLT-Seq libraries are first processed through cutadapt (v3.7)^32^ to isolate and remove anchoring sequences from the barcode structure (Figure 5, cutadapt step). Next, CITE-seq-count is used to build a count table of the number of barcodes identified per cell (Figure 5, CITE-seq-count step),^33^ data that is further processed using R to binarize the counts table. After binarization, processed data solely displays presence/absence of a given barcode per cell and can be used to identify, estimate and filter over-represented barcodes by setting a threshold of number of cells harboring the same barcode (empirical or experimental). Finally, a similarity score is calculated following the code published along with the original article^1^ that establish the number of barcodes that a cell has in common with any other cell present in the subsequent sample time point. The similarity score table provides the basis to build the lineage tree.

In parallel to BdLT-Seq data analysis, 10× Chromium single-cell and HTO sequencing data is processed by Seurat (v4 or later) using standard pipelines^34^^,^^35^ to demultiplex samples (time points), for quality control and cell masking (nFeature_RNA, nCount_RNA and mitochondrial content). Further pre-processing includes the mathematical regression of cell cycle and mitochondrial content bias prior to clustering.^34^^,^^35^

Once all the sequencing datasets have been pre-processed (BdLT-Seq, HTO and GEx), datasets are matched to analyze transcriptome divergence over time and lineage tracing embedded relationships between tree branches. Gene expression states can be represented using CIRCOS plots.^36^ Final data presentation is to be processed in Adobe Illustrator if required.

***Note:*** Processing the whole BdLT-Seq library through CITE-Seq-count is computationally demanding even using an HPC system. We suggest the end-user to random sample the number of barcodes piped into CITE-Seq-count down to 100 K–150 K barcodes per experiment to optimize computational time. We have observed that increasing the number of barcodes analyzed further does not impact on BdLT-Seq lineage outcomes and only mild improvement has been observed (∼1–2 cells every 100 K additional barcodes analyzed).

## Limitations

BdLT-Seq has a few limitations that must be considered before adopting the method.

BdLT-Seq relies on the generation of sibling cells through cell division. Each of these sibling cells inherits a subset of barcoded episomes from the ancestor cell in a stochastic manner. These characteristics enable the sampling of descendants from each lineage and reconstruction of lineage trees based on barcode similarity. Consequently, BdLT-Seq is not applicable to non-proliferating or dormant cells. Additionally, due to the aforementioned reasons, transcriptome dynamics occurring within the same cell cycle cannot be explored.

With a relatively low probability, stable integration of the plasmid into the genome may occur. Given the infrequent nature of these events and the relatively short duration of BdLT-Seq experiments, we do not consider this to be a significant concern. Furthermore, biological replicas of the BdLT-Seq experiments did not reveal any significant alterations or fluctuations in non-genetic heterogeneity as determined by scRNA-Seq.

BdLT-Seq high-complexity episomal libraries can accommodate up to ∼16.7 x 10^6^ unique barcodes and are transfected into cells via standard protocols. Since almost every episome in our library harbors a unique barcode, the combination of episomes that is introduced into any given cell generates a molecular fingerprint allowing the identification of each cell within the population. The number of barcode combinations introduced to any given cell can reach extremely high numbers, meaning that millions of cells and their lineages could be analyzed simultaneously. Furthermore, we can assign identity to 85%–90% of the cells analyzed. However, a major limitation of BdLT-Seq relies on sequencing costs. This limitation leads to a significant reduction in the number of lineages that can be captured and analyzed. Thus, careful experimental planning and assessment of the starting number of cells/lineages is absolutely essential for the optimal performance of BdLT-Seq (highest possible number of lineages traced to the lowest possible sequencing cost).

## Troubleshooting

### Problem 1

Low number of colonies generated (related to steps 7–8).

### Potential solution

• Avoid heat-inactivating the ligation reaction before bacterial transformation, as this can substantially decrease transformation efficiency.
• Avoid processing too many bacteria transformations simultaneously. The time taken both before and after bacteria electroporation is critical for optimal recovery post electroporation. Spending a prolonged amount of time before and after electroporation will significantly reduce transformation efficiency.
• Utilize a substantial amount of S.O.C. medium for post-electroporation recovery. The use of 4–5 mL of S.O.C. medium upon electroporation could potentially enhance transformation results.
• We have observed significant variability in the transformation potential of electrocompetent bacteria across different batches. Therefore, it may be necessary to switch to a different batch of bacteria to improve transformation efficiency.

### Problem 2

High barcode multiplicity of the BdLT-Seq library. The BdLT-Seq episome backbone harbors a low copy origin of replication based on *bom rop* and is only functional in bacteria. Nevertheless, caution should be taken to minimize the emergence of episomes containing barcodes harboring the same sequence (related to step 8).

### Potential solution

• The plates generated by seeding undiluted bacteria are meant for collection and plasmid extraction (step 8). Plating between 30 K–300 K bacteria per plate inhibits bacteria proliferation and thus colony growth, thereby reducing the number of episome copies per colony and therefore reducing the number of identical barcodes. Keeping the multiplicity of barcodes low is crucial for lineage tracing experiments.
• Do not allow the bacteria to overgrow. Collect the colonies from undiluted plates when small colonies are observed. If necessary, the temperature of the overnight incubation can be reduced to 30°C to slow down colony growth.

### Problem 3

Low transfection efficiency (related to steps 18–20).

### Potential solution

• Optimal transfection conditions may need to be established for a specific cell line of interest. Experiment with various amounts of Plus reagent and Lipofectamine LTX reagent to optimize transfection efficiency. Alternatively, consider trying different transfection reagents (e.g., Lipofectamine 2000, etc.) or different transfection methods (e.g., nucleofection).

### Problem 4

Low post-sort purity of the sample (related to step 20).

### Potential solution

• We recommend configuring the sorter for your cell type. Establishing a suitable drop delay for the specific cell line often contributes to obtaining optimal purity results.

### Problem 5

Cells do not recover well after FACS (related to steps 20–21).

### Potential solution

• We recommend configuring the sorter for your specific cells. Often, adjusting the pressure configuration to 16 psi enhances cell recovery. Modifying the nozzle size could also potentially improve cell recovery.

### Problem 6

Low number of lineages traced.

### Potential solution

• The initial cell count is crucial for capturing and tracing the maximum number of lineages. We recommend starting with no more cells than what will ultimately be sequenced. Please refer to step 22 for more details.

### Problem 7

Clumpy cells (related to steps 23 d and i). It is critical to ensure that single cells are easily distinguishable and that no clumps or debris are present.

### Potential solution

• If cell clumps are observed, you can filter the cell suspension. Use a filter with pore size that fits the size of the cells. Note that filtering may lead to a reduction in cell count.

### Problem 8

Low efficiency of H2B-GFP cDNA capturing (related to step 29).

### Potential solution

• We recommend verifying in advance that biotinylated capture probes indeed work as intended.
• We suggest purifying the biotinylated probes before using them for BdLT-Seq. For more details, please refer to ‘Preparation of sequence-specific biotinylated probes against H2B-GFP’ at the beginning of this protocol.

### Problem 9

The TSO-RT-oligo product (∼150 bp) can be amplified during the HTO PCR by carryover primers from the cDNA amplification reaction. This PCR product will not cluster during sequencing but will interfere with library quantification (related to step 33).

### Potential solution

• Re-run the HTO PCR amplification by increasing cycling by 1–2 additional cycles and assess the library quality using a Fragment Analyzer.

## Resource availability

### Lead contact

Further information and requests for resources and reagents should be directed to and will be fulfilled by the lead contact, Maximiliano Portal (Maximiliano.Portal@glasgow.ac.uk).

### Technical contact

For technical questions and advice, please contact Yelyzaveta Shlyakhtina (lisaschliakhtina@gmail.com), Bianca Bloechl (b.blochl@beatson.gla.ac.uk), or Maximiliano Portal (maximiliano.portal@glasgow.ac.uk).

### Materials availability

BdLT-Seq episome (LT_vector) can be requested by contacting Maximiliano Portal.

### Data and code availability

BdLT-Seq data is to be analyzed using publicly available, benchmarked, and validated pipelines (R v4.2.0, Seurat v4.0, CITE-seq-count and cutadapt v3.7)^32-35^. Data analysis rationale is described in quantification and statistical analysis section and illustrated in Figure 5. A Custom Node script to determine barcode similarity (lineage relationships) is provided within the original research manuscript.^1^ For data analysis related inquiries contact M.M.P. (Maximiliano.Portal@glasgow.ac.uk).

## Supplemental information

Table S1. Primers for construction of NEBNext libraries, HTO libraries, and HTO additive
