# Supplementary material for: Protocol to study the inheritance and propagation of non-genetically encoded states using barcode decay lineage tracing
Source: STAR Protoc. 2024 Jan 4;5(1):102809. doi: 10.1016/j.xpro.2023.102809 (PMC10801334; doi:10.1016/j.xpro.2023.102809)
Supplement: Table S1. Primers for construction of NEBNext libraries, HTO libraries, and HTO additive [file mmc1.pdf]

Supplementary Table S1: Primers for construction of NEBNext libraries, HTO libraries and HTO additive, related to Key resources table, step 15, step 25 and step 33.

Oligonucleotides		
NEBNext_universal_PCR_primer_for_Illumina AATGATACGGCGACCACCGAGATCTACACTCTTTCCCTACACGACGCTCTTCCGATCT	NEB	E7335
NEBNext_index_primer_for_Illumina CAAGCAGAAGACGGCATACGAGATNNNNNNGTGACTGGAGTTCAGACGTGTGCTCTTCCGATCT	NEB	E7335
HTO_additive GTGACTGGAGTTCAGACGTGTGCTCTTCCGATCT	Merck	N/A
HTO_library_Forward primer_BC1 CAAGCAGAAGACGGCATACGAGATCGAGTAATGTGACTGGAGTTCAGACGTGTGC	Merck	N/A
HTO_library_Forward primer_BC2 CAAGCAGAAGACGGCATACGAGATTCTCCGGAGTGACTGGAGTTCAGACGTGTGC	Merck	N/A
HTO_library_Forward primer_BC3 CAAGCAGAAGACGGCATACGAGATAATGAGCGGTGACTGGAGTTCAGACGTGTGC	Merck	N/A
HTO_library_Reverse primer AATGATACGGCGACCACCGAGATCTACACTCTTTCCCTACACGACGCTC	Merck	N/A
